# CXCR4 Coordinates Long-Term Olfactory Epithelial Regeneration and Cellular Stress Response After Injury

**DOI:** 10.1007/s12035-026-06215-x

**Published:** 2026-09-23

**Authors:** Katja Senf, André Dietz, Julia Karius, Eva M. Neuhaus

**Affiliations:** 1 Pharmacology and Toxicology, Jena University Hospital, Friedrich Schiller University Jena, Drackendorfer Str. 1, 07747 Jena, Germany; 2 Center for Sepsis Control and Care, Department of Anesthesiology and Intensive Care, Jena University Hospital, Jena, Germany

**Keywords:** CXCR4, Olfactory, Axon guidance, Horizontal basal cell, NQO1, Stress response

## Abstract

During development, the chemokine receptor CXCR4 directs axon guidance, but it is not known if CXCR4 regulates axon targeting in the adult organism as well. We utilized the remarkable feature of the olfactory epithelium to replace damaged neurons throughout life to understand the role of CXCR4 in adult neurogenesis. Through targeting *Cxcr4* in horizontal basal stem cells of the olfactory epithelium, we investigated the role of CXCR4 during injury-induced neurogenesis in adult mice by a combination of RNA-seq analysis, bioinformatic methods, behavioral experiments, and microscopical approaches. Conditional knockout mice lacking *Cxcr4* initially displayed enhanced regeneration after injury of the olfactory system, resulting in increased numbers of sensory neurons and enhanced sense of smell. Moreover, we found that absence of CXCR4 alters expression of axon guidance proteins. Despite accelerated early neurogenesis, loss of CXCR4 ultimately impaired the reconstruction of the dorsomedial olfactory epithelium, leading to disrupted glomerular organization and reduced avoidance responses to aversive odors. Transcriptomic analysis revealed altered stress response pathways, suggesting impaired cellular adaptation in *Cxcr4*-cKO mice during regenerative repair. An increased level of ER stress in horizontal basal cells led to instability of the basal lamina, respiratory metaplasia in the dorsomedial part of the olfactory epithelium, and Bowman gland regeneration failure.

**Supplementary Information:**

The online version contains supplementary material available at https://doi.org/10.1007/s12035-026-06215-x.

## Introduction

To promote functional recovery in the adult mammalian central nervous system, a better understanding of the molecular mechanisms is needed that enables coordinated neuronal tissue repair, long-distance axon regeneration, and re-establishment of neuronal circuits. The expression pattern of specific transcription factors selects combinations of receptors for attraction/repulsion molecules, cell–cell contact proteins, and molecules involved in synaptic partner matching going to be expressed on the cell surface and allowing the neurons to make proper connections.

The olfactory epithelium is a unique model to study neurogenesis and regeneration, as it continuously replaces OSNs (olfactory sensory neurons), which reside in the nasal cavity and project their axons to the olfactory bulb. OSNs are responding to odors in the environment and transmit the information to higher brain centers. Neuronal identity is determined by the stochastic choice of one olfactory receptor out of more than 1000 receptors in the mouse genome, and the choice of the receptor determines the connection in the olfactory bulb [1]. All neurons expressing the same receptor target a specific glomerulus or a small set of glomeruli; olfactory receptor identities therefore are essential for proper axonal projections. The mechanism by which G protein-coupled olfactory receptors are converted into the guidance molecules required for proper glomerular segregation involves cAMP signaling [2, 3], as well as the regulation of the unfolded protein response [4]. In addition, odorant receptors at the axon terminals could respond to molecules originating in the olfactory bulb thereby acting as axon guidance cues [5]. Despite the remarkable regenerative capacity of the olfactory epithelium, the molecular mechanisms that coordinate neuronal replacement with accurate circuit reconstruction remain incompletely understood.

The G-protein coupled receptor CXCR4 (CXC-motif chemokine receptor 4) plays a role in neurogenesis and axon pathfinding in different neuronal tissues during development. Its ligand CXCL12 (CXC-motif chemokine ligand 12) is a repulsive cue for efferents of rat cerebellar neurons [6] but also an attractant for other axons such as retinal ganglion cells [7] and OSNs in zebrafish [8]. CXCL12 reduces the repellent activities of SLIT-2 on cultured retinal ganglion cell axons, of semaphorin 3 A on dorsal root ganglion sensory axons, and of semaphorin 3 C on sympathetic axons [9]. Segregation of retinal ganglion cell axons at the optic chiasm depends on CXCL12 being expressed by the meninges bordering the optic pathway and on expression of CXCR4 [10]. Axonal CXCR4 and CXCL12 secreted by tissues surrounding the neural tube are required for ventral motor neurons to establish the essential connection between brain and periphery [11]. Despite the multiple studies showing the importance of CXCR4 during developmental axon pathfinding, little is known if CXCR4 also contributes to adult neurogenesis. In the adult olfactory epithelium, CXCR4 is expressed by immediate neuronal progenitor cells and immature OSNs and plays a role in olfactory neurogenesis [12, 13]. CXCL12 is expressed in the lamina propria of the olfactory epithelium [14] and therefore might serve as a chemoattractant that guides axons of OSNs to the bulb or could create a permissive environment for axon outgrowth.

We here investigate the role of CXCR4 during injury-induced regeneration of OSNs from adult stem cells. Combining transcriptomic analysis, molecular profiling, histological approaches, and behavioral assessment, our findings identified CXCR4 as a critical regulator of proper axon extension and accurate targeting of their axons to their appropriate glomeruli in the olfactory bulb. Moreover, we found that absence of CXCR4 alters the molecular properties of stem cells and ultimately impairs proper regeneration of the dorsomedial parts of the olfactory epithelium.

## Material and Methods

### Experimental Models

Animal experiments were conducted in accordance with the EC directive 86/609/European Economic Community guidelines for animal experiments and permitted by the local government (Thüringer Landesamt für Lebensmittelsicherheit und Verbraucherschutz). Mice were kept under 12-h light/dark cycles with ad libitum access to food and water. C57BL/6J wild-type mice and Tg(*Krt14-Cre*)1Amc mice (#004782 [15]) were purchased from Charles River Laboratories (Sulzfeld, Germany). *Krt14* is expressed in horizontal basal cells of the olfactory epithelium [16]. Expression of tdTomato was used to control for the expected localization of promoter activation (Tg(*Krt14*-cre);R26^CAG-LSL-tdT^). *Cxcr4*^LoxP/LoxP^ mice [17] were on a C57BL/6J background. Tg(*Krt14*-cre);*Cxcr4*^*LoxP*/*LoxP*^ mice were generated in order to delete *Cxcr4* in *Krt14*-expressing horizontal basal cells. For the regeneration experiments, mice at 2 m.o. (month of age) or 18 m.o. were injected intraperitoneally with either 50 mg/kg methimazole or 0.9% sodium chloride (control). Mice were euthanized with an overdose of isoflurane (5%) at 1, 3, 14, 28, and 42 days post-injection (dpi). Mice of both sexes were used for the experiments.

### Tissue Preparation

To prepare the olfactory epithelium, vomeronasal bones, zygomatic bones and plate, nasal bones, the lower jaw, incisors, and the anterior maxillae were removed according to a modified protocol [18]. For immunofluorescence, the olfactory epithelium was fixed in 4% paraformaldehyde for 24 h at 4 °C, cryopreserved in 30% sucrose in PBS (phosphate-buffered saline) for at least 24 h at 4 °C, and frozen in 2-methylbutane (Carl Roth, Karlsruhe, GER). For in situ hybridization, the olfactory epithelium was frozen in 2-methylbutane without prior fixation. The tissue samples were embedded in tissue freezing medium (Leica, Germany) and sectioned coronally at 18-µm thickness. For quantitative PCR, tissue samples were frozen in liquid nitrogen.

### Immunofluorescence

In brief, slides were washed in Tris-buffered saline (TBS), incubated in citrate buffer (0.1 mol/l trisodium citrate dihydrate, 0.5% Tween-20, pH 6) at 97–99 °C for 15 min to unmask epitopes, and blocked with TBS Plus (2% bovine serum albumin, 3% normal donkey serum, 0.1% Triton in TBS) for at least 1 h. The slides were incubated with the primary antibody (Supplementary Table S1) overnight at 4 °C. After washing, the slides were incubated in the secondary antibody solution for 2 h at room temperature (1:500 in TBS Plus, Supplementary Table S2). The detection of aggresomes was performed with Proteostat® Aggresome Kit (ENZ-51035, Enzo Life Sciences GmbH, DE-79540 Lörrach, Germany) as recommended in the manufacturer’s protocol. Aggresomes are inclusion bodies of aggregated, misfolded proteins that form in response to cellular stress. Slides were incubated for 30 min at room temperature in assay buffer with Proteostat® aggresome detection reagent (1:500) and Hoechst (1:1000), followed by washing in PBS.

### 3,3-Diaminobenzidine (DAB) Staining

After washing in TBS and epitope unmasking in 0.1 mol/l citrate buffer (97–99 °C for 15 min), slides were incubated in 0.3% hydrogen peroxide and washed in TBS. Slides were blocked in TBS Plus for 1 h and incubated with the primary antibody solution at 4 °C overnight (Supplementary Table S1). After washing in TBS, the slides were incubated with a biotinylated secondary antibody for 2 h at room temperature (Supplementary Table S2), followed by the avidin–biotin complex for 1 h. Slides were stained with 0.7 mg/ml DAB in combination with 1.6 mg/ml hydrogen peroxide in 0.06 M Tris buffer.

### High Iron Diamine (HID)–Alcian Blue Staining

To characterize the production of acidic mucins by the Bowman’s glands, high iron diamine (HID)–Alcian Blue staining was performed. HID staining predominantly labels sulfated acidic mucins, resulting in a dark brown to black staining; Alcian Blue labels nonsulfated acidic mucins [19, 20]. The HID solution was prepared by dissolving 120-mg N,N-dimethyl-m-phenylenediamine dihydrochloride and 20-mg N,N-dimethyl-p-phenylenediamine dihydrochloride in 50-ml distilled water supplemented with 1.4-ml 10% ferric chloride solution. The slides were incubated in the HID solution for 18 h under constant agitation in the dark, washed in TBS, and incubated in Alcian Blue for 2 min.

### NADPH Diaphorase Assay

For the NADPH diaphorase assay, slides were washed in 100 mM Tris–HCl pH8 und incubated for 2 h at RT in 1 mM β-nicotinamide adenine dinucleotide phosphate (NADPH), 0.8 mM nitroblue tetrazolium chloride (NBT) and 0.3% Triton X-100 (Merck Millipore, Darmstadt, GER) according to a modified protocol [21]. Afterwards, slides were washed in Tris–HCl, dehydrated, and mounted with Entellan™.

### In Situ Hybridization

Fluorescent in situ hybridization was performed on 20-µm cryosections of olfactory epithelium from 2 m.o. mice. Sections were fixed in ice-cold 4% paraformaldehyde for 1 h and permeabilized in 0.4% Triton X triethanolamine buffer (pH 8) and acetic anhydride. For probe construction, mouse *Atf5* cDNA was amplified from murine olfactory epithelium mRNA by PCR and cloned into pcDNA3 (amplified region 299–1151 nt; 853 bp; gene ID 107503; NM030693). The riboprobe for the detection of *Perk* (*Eif2ak3*) transcripts was generated using the plasmid PERK.WT.9E10 in pCDNA as a gift from David Ron [22] (Addgene, #21814). For hybridization, sections were incubated with 2 µg/ml *Atf5* or *Perk* riboprobe in hybridization buffer for 20 h at 60 °C in a humid chamber containing 50% formamide. Sections were washed in 1 × SSC (trisodium citrate dihydrate) at room temperature, incubated in RNAse solution for 30 min at 42 °C, and washed again in 0.2 × SSC. Sections were transferred into maleic acid and incubated in blocking buffer for 1 h at room temperature. Sections were incubated in anti-digoxigenin-POD Fab fragments (1:500, 30 min), washed in maleic acid, and incubated with TSA™ Plus Cyanine 3 Amplification Kit (Akoya Biosciences, Cat# NEL704A001KT). Slides were mounted with Fluoromount-G™ (Thermo Fisher Scientific).

### Microscopy and Quantification of Microscopical Images

Brightfield images were taken using a Zeiss Axio Imager A1 microscope (Carl Zeiss Meditec, Jena, GER) equipped with Progres Gryphax Arktur camera (Jenoptik AG, Jena, GER). Fluorescence was detected with a confocal laser scanning microscope with TCS SPE system (Leica DM2500, Leica Microsystems, Wetzlar, GER) or with a Zeiss LSM900 equipped with Airy-Scan technology (Carl Zeiss Microscopy GmbH, Oberkochen, GER).

For quantification of immunofluorescent images, same-sized Z-stacks were selected and four images per region (D-zone, V-zone, Fig. S1) were analyzed in 3–6 animals per group. Four to six cryosections were quantified and averaged. Sections were stained in parallel, and images taken for quantification were taken with the same microscope (TCS SPE system or Zeiss LSM900). Measurements of areas and cell counts were performed on digital pictures using LAS X, ImageJ, or ZEN 3.0. Cell counts were normalized to 1-mm OE. Fluorescence intensities were normalized to the background; the background intensity was determined in cells that do not express the protein of interest based on visual inspection. Data from each animal were averaged and used for statistical analysis; a minimum of three different mice were analyzed per condition. Images were further processed using LAS AF (Leica Microsystems), ImageJ, Photoshop CS6 (Adobe Systems, CA, USA), and ZEN 3.0 (blue edition) (Carl Zeiss Microscopy GmbH, Oberkochen, GER). Statistical analysis was performed in GraphPad Prism 5.01; data were represented as mean ± SEM. Data were tested for normal distribution and homogeneity. Statistical significance was set at *probability (*p*) < 0.05 and analyzed using Student’s *t* test.

### Quantitative Real-Time PCR

Total RNA from 2 m.o. wild-type (WT) and *Krt14-Cre; Cxcr4*^*loxP/loxP*^ mice was isolated from olfactory mucosa samples using Purelink RNA Mini Kit (Thermo Fisher Scientific, Cat# 12183018 A); cDNA was generated with High Capacity cDNA Kit (Thermo Fisher Scientific, Cat# 4368814). Quantitative real-time PCR (qPCR) was performed on a Quant Studio® 3 Real-Time PCR Cycler (Thermo Fisher Scientific Germany Ltd. & Co. KG, Bonn, GER) using predesigned Quantitect primers (Qiagen) for *Gapdh*, *Ano2*, *Ascl1*, *Atf5*, *Cnga2*, *Exoc4*, *Hspa5*, *NQO1*, *Olf16*, *Olfr545*, *OMP* and Power Up SYBR® Green Master Mix (Thermo Fisher Scientific, Cat# A25778). qPCR conditions were 2 min 50.0 °C, 2 min 95.0 °C denaturation, and 44 cycles consisting of 15 s 95 °C, 1 min 55 °C. At the end of the cycles, the sample was kept at 10 °C. Three independent runs were performed with each sample. Expression levels of mRNA were determined using the ΔΔCT method and displayed as 2^−ΔΔ*CT*^ values [23].

### RNA Sequencing and Transcriptomic Analysis

Main olfactory epithelium was collected from 8 m.o. WT and *Krt14*-Cre;*Cxcr4*^LoxP/LoxP^ mice (three animals per genotype). RNA isolation was done using Purelink RNA Mini Kit; RNA concentration and purity were determined by NanoDrop Lite (Thermo Fisher Scientific Germany Ltd & Co. KG, Bonn, GER). RNA integrity was controlled on denaturing agarose gels. Transcriptional sequencing and DE analysis were performed by Eurofins Genomics Germany GmbH (Ebersberg, GER); the dataset was filtered for an adjusted *p* value cutoff of 0.05. To determine the cellular localization and average expression, differentially expressed genes were identified in a published RNA single cell-sequencing dataset GSE169011 [24]. Visualization was done using the DotPlot function of the R software package Seurat V4 [25]. Gene ontology enrichment and network analysis was accomplished using ShinyGO 0.76.3 [26] for GO biological processes and FDR cutoff of 0.05.

### In Silico Perturbation Analysis Using CellOracle

Perturbation analysis, including lineage tracing as a prerequisite, was performed using the Python library CellOracle [27]. CellOracle version 0.14.0 was installed in an Anaconda environment running Python 3.8 on an ARM MacBook Pro with macOS Sonoma 14.0. The installation was performed using the GCC compiler and Xcode command line tools, following the guidelines provided in the CellOracle documentation. The web-based Jupyter Notebook application was utilized for code execution. We utilized the publicly available single-cell RNA sequencing dataset GSE169011, representing the main olfactory epithelium [24], to generate a neuronal subset comprising the cell identities INP, iOSN, and OSN. This neuronal subset dataset was exported as a matrix file using the R software package DropletUtils 1.18.1 [28] and subsequently loaded into CellOracle. For clustering and UMAP projection, parameters were set to n_neighbors = 20 and n_pcs = 10. The cell identity V3M1_TCGAACATCGATACAC was designated as the root cell for pseudotime calculation. For creation of a custom gene regulatory network (GRN) for the adult main olfactory epithelium, we used the published single-nucleotide transposase-accessible chromatin sequencing (snATACseq) fragments file GSM7159487 [29]. The fragments file was processed with the R package Signac 1.12.0 [30] and converted into matrix, peaks, and barcode files with parameters set to a total counts cutoff value of > 1000, min.cells = 10, and min.features = 200. Gene annotations were based on the Ensembl “EnsDb.Mmusculus.v79” database [31]. Further processing and formatting to generate the main olfactory epithelium base GRN were performed using the R package Cicero 1.3.9 [32] and Python 3.8, according to the CellOracle documentation for input data preparation. The perturbation analysis was conducted following the standard workflow outlined in the CellOracle documentation.

### Cell Type Deconvolution from Bulk RNA Data Using CIBERSORTx

For in silico identification of different cell types within bulk RNA sequencing datasets, the analytical tool CIBERSORTx was used [33]. To calculate the abundance for each cell type of the main olfactory epithelium represented in our bulk RNA data, a main olfactory epithelium-specific signature matrix file was generated (Cibersort supplements). The resulting file comprised uniquely expressed barcode genes for each cell type to serve as a template to impute cell fractions within bulk RNA data. Additionally, a source gene expression profile (sourceGEP) file for batch correction was built by CIBERSORTx during this process. The signature matrix file and sourceGEP file were built from a public single-cell RNA sequencing dataset of the main olfactory epithelium (GSE169011) [24]. Using the “subset” function of the R module Seurat 5.0.1 [34], the dataset was down-sampled to 100 cells for each cell type identity. Subsequently, the RNA counts for each gene in each cell were exported and provided to the CIBERSORTx application as a single-cell reference sample file (Cibersort supplements). For calculation of the cell type barcode genes, the input data type was set to “scRNA-Seq” and recommended presets were used. Determination of cell types in each bulk RNA expression sample was done utilizing the “Impute Cell Fractions” analysis module. Normalized sample-wise gene read counts for each condition and genotype, reflecting the mixed cell population for each tested animal, were provided as mixture file input. The main olfactory epithelium-specific signature file along with the sourceGEP file was used for cell type deconvolution in absolute mode including enabled batch correction in B-mode and disabled quantile normalization.

### Behavioral Tests

To increase the motivation for food searching behavior, the mice were not fed for 18 h before the cookie finding test. WT and Krt14-Cre; CXCR4^loxP/loxP^ mice (*n* = 9–21/time point and genotype, 81 mice in total) were placed in a clean cage with bedding for a 5-min habituation phase; afterwards, a Froot Loop® was hidden in the bedding and mice were placed back in the cage. For 900 s (15 min), the time to find the cookie was recorded. Latency and hit rate (found or not in 900 s) were measured.

For the avoidance test at 42 dpi, WT and *Krt14*-Cre; *Cxcr4*^loxP/loxP^ mice (*n* = 9–12/time point and genotype, 24 in total) were transferred into the test cage including a Petri dish. The behavior was recorded with a fixed video camera on top. At the beginning, 100-µl water as a control was dropped on a filter paper in the petri dish and the behavior was recorded for 3 min. Afterwards, 100-µl hexanal (1:10) was added on a filter paper and placed into the cage. The behavior was recorded again for 3 min. Movements were analyzed using the free ICY Software Mice Profiler Tracker [35].

## Results

### CXCR4-cKO in Horizontal Basal Cells Causes Mild Phenotypic Changes Under Homeostatic Conditions

CXCR4 is expressed by globose basal cells, immediate neuronal progenitors, and immature neurons [13] and is equally distributed along the anterior posterior axis of the olfactory epithelium (Fig. S2a). The olfactory epithelium contains two progenitor populations. Under homeostatic conditions and during development, the differentiation of actively proliferating globose basal cells sustains olfactory neurogenesis [36–38]. Horizontal basal cells do not contribute significantly to developmental or homeostatic neurogenesis but form sustentacular cells, supporting cells of the olfactory epithelium [39]. Horizontal basal cells are quiescent stem cells that appear in the epithelium only after birth but become activated to repair the system after severe damage with loss of differentiated cells and globose basal cells [40–42]. To specifically investigate the role of CXCR4 in adult neurogenesis and not during development, we deleted the gene in horizontal basal cells to study injury-induced neurogenesis. KRT14 is a marker for horizontal basal cells throughout the olfactory epithelium (Fig. S2b) [16, 43]. We therefore generated transgenic mice expressing Cre-recombinase under the control of the *Krt14* (Keratin 14) promoter (Tg(*Krt14*-cre)1Amc;*Cxcr4*^Loxp/LoxP^, named *Cxcr4*-cKO) to delete *Cxcr4* in horizontal basal cells.

**Fig. 1:**
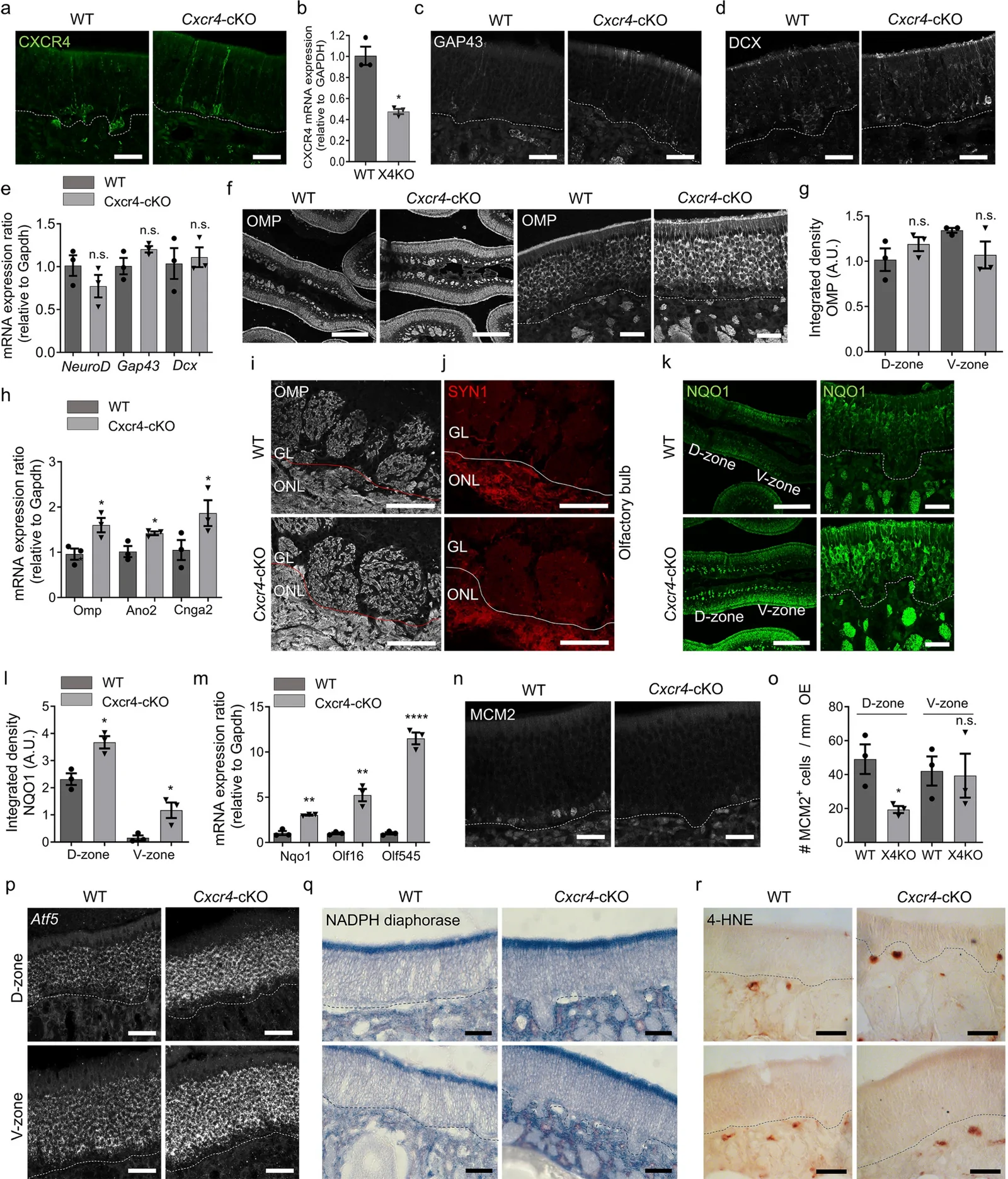
Reduced CXCR4 levels are linked to mild phenotypic changes under homeostatic conditions. **a** CXCR4 expression was not significantly reduced in Cxcr4-cKO mice, as demonstrated by immunofluorescence analysis in the D-zone of the epithelium. **b** Quantitative PCR from olfactory epithelium mRNA isolated from adult WT and *Cxcr4*-cKO animals showed a reduction of *Cxcr4* by around 50% in knockout mice. Immunofluorescent staining for GAP43 (**c**) and DCX (**d**) exhibited no obvious differences between WT and *Cxcr4*-cKO in the D-zone of the olfactory epithelium. **e** Quantitative PCR from olfactory epithelium mRNA isolated from adult WT and *Cxcr4*-cKO animals. There was no significant difference comparing markers for neuronal precursor cells (NeuroD) and for immature neurons (Gap43, Dcx) between WT and *Cxcr4*-cKO animals. **f** Left Overview image of olfactory marker protein (OMP) in the olfactory epithelium showing equal distribution of OMP in WT and *Cxcr4*-cKO. **Right** Higher magnification of the D-zone showing immunolabeling of OMP is slightly increased in *Cxcr4*-cKO mice. **g** Quantification of OMP staining intensities did not show significant differences between WT and *Cxcr4*-cKO mice. **h** Quantitative PCR from olfactory epithelium mRNA isolated from WT and *Cxcr4*-cKO animals at steady state. Expression of ubiquitous mature OSN markers *Omp*, *Ano2*, and *Cnga2* was increased in *Cxcr4*-cKO. Immunofluorescent labeling of OMP (**i**) and SYN1 (synaptophysin 1, **j**) in the olfactory bulb of adult WT and *Cxcr4*-cKO mice. OMP was clearly visible in the outer nerve layer and within the round structured glomeruli in both, WT and *Cxcr4*-cKO. There was also no difference in SYN1 labeling, indicating normal axonal outgrowth and targeting in *Cxcr4*-cKO mice. Lines represent the border between outer nerve layer (ONL) and glomerular layer (GL). **k** Immunostaining of NQO1, a marker for neurons in the D-zone, was more intense in *Cxcr4*-cKO animals and the staining was expanded into the ventral part (V-zone) of the olfactory epithelium. **l** Quantification of NQO1 staining intensities, showing a significant increase of NQO1 in *Cxcr4*-cKO animals. **m** Quantitative PCR from olfactory epithelium mRNA isolated from WT and *Cxcr4*-cKO animals at steady state. Expression of D-zone markers *Nqo1* and olfactory receptors *Olfr16* and *Olfr545* was increased. **n** In *Cxcr4*-cKO mice, there were fewer proliferating cells stained with MCM2 (minichromosome maintenance protein 2). **o** Quantification of MCM2-positive cells in the D-zone and the V-zone of the olfactory epithelium. Proliferation was significantly reduced in the D-zone of *Cxcr4*-cKO mice compared to WT, without differences in the V-zone. **p** In situ hybridization for *Atf5* in noninjured epithelium showing increased *Atf5* expression in *Cxcr4*-cKO mice compared to WT in the D-zone and V-zone of the epithelium. **q** NADPH diaphorase labeling, a histochemical marker for nitrosative stress showing NADPH-dependent reduction of nitroblue tetrazolium to diformazan. **r** 3,3-Diaminobenzidine (DAB) labeling of 4-HNE showing no obvious differences between WT and *Cxcr4*-cKO. Scale bars: **a**, **c**, **d**, **f** right, **k** right, **n**, **p**, **q**, **r** 20 μm. **f** left, **k** left, **i**, **j** 50 µm. Dotted lines represent the basal lamina of the epithelium. Quantification of *n* = 3 animals per group, Student’s *t* test, error bars represent SEM. ^*^p < 0.05, ^**^p < 0.01, ^***^p < 0.001, and ^****^p < 0.0001

Before investigating injury-induced regeneration, we started by analyzing the phenotype of the noninjured mice at steady state. Immunofluorescence staining revealed that *Cxcr4*-cKO did not significantly affect CXCR4 expression in any region of the olfactory epithelium (Fig. 1a, Fig. S2c). This was expected, since *Cxcr4* is predominantly expressed in proliferating globose basal cells [13] which do not express *Krt14*. Despite similar protein expression, quantitative PCR of the olfactory mucosa revealed a reduction of *Cxcr4* mRNA in *Cxcr4*-cKO mice compared to WT (wild-type) controls under steady-state conditions (Fig. 1b).

Protein and mRNA levels of neuronal precursors (*NeuroD* (neuronal differentiation 1)) and immature neurons (*Gap43* (growth-associated protein 43), *Dcx* (doublecortin)) were not different between WT and *Cxcr4-*cKO (Fig. 1c-–e, Fig. S2d, e). Staining of mature neurons with OMP (olfactory marker protein) appeared slightly increased in *Cxcr4-*cKO mice (Fig. 1f, Fig. S2f). However, quantification did not reveal any significant differences (Fig. 1g), probably because of the high overall levels of OMP expression, which prevented small differences from being detected by immunohistochemistry; mRNA expression levels of *Omp* and other markers for mature OSNs, *Ano2* (anoctamin 2) and *Cnga2 *(cyclic nucleotide gated channel A2), were increased (Fig. 1h). Immunofluorescent staining of the olfactory bulb with OMP and SYN1 (synaptophysin 1) revealed no obvious differences in glomerular organization in *Cxcr4-*cKO mice (Fig. 1i, j).

The olfactory epithelium is divided into distinct spatial zones with specific odorant receptor genes and other molecular marker patterns. The enzyme NAD(P)H quinone oxidoreductase 1 (NQO1) is selectively expressed by mature sensory neurons located within the dorsomedial region (D-zone, but not in neurons in the ventral part (V-zone) of the olfactory epithelium [44] (Fig. S1). Interestingly, *Cxcr4-*cKO mice exhibited elevated levels of the D-zone marker protein NQO1 and an expansion of NQO1-positive cells in the ventral region, when compared with WT animals (Fig. 1k, l, Fig. S2g). Increased abundance of dorsomedial neurons was also reflected by increased expression of *Nqo1* mRNA and olfactory receptors *Olfr16* (olfactory receptor 16) and *Olfr545* (olfactory receptor 545) (Fig. 1m)*,* known to be specifically expressed in the D-zone of the epithelium [45, 46]. Moreover, cell proliferation activity also showed regional differences. While the number of MCM2 (minichromosome maintenance complex component 2)–positive cells was similar in the V-zone, we detected a significant reduction of cycling cells in the D-zone of *Cxcr4*-cKO mice (Fig. 1n, o, Fig. S2h). Together with the increased expression of *Olfr16*, *Olfr545*, and *Nqo1*, this indicates increased differentiation of neurons in the D-zone.

As NQO1 is involved in antioxidant responses, we next examined the expression of the transcription factor ATF5 (activating transcription factor 5) that is involved in cellular differentiation and promotes cellular adaptation to stress [47]. Higher levels of *Atf5* expression indicated elevated stress levels in *Cxcr4*-cKO mice (Fig. 1p). ATF5 is also important for maturation and survival of OSNs [48]; increased expression may also explain the slight increase in mature OSNs. Moreover, we found increased labeling of NADPH (nicotinamide adenine dinucleotide phosphate diaphorase) in the olfactory epithelium of *Cxcr4*-cKO mice (Fig. 1q). Increased NADPH diaphorase labeling indicates increased redox signaling and indicates elevated levels of either oxidative stress or ER (endoplasmic reticulum) stress [49]. However, since antibody staining against the lipid peroxidation marker 4-HNE (4-hydroxynonenal) did not reveal major oxidative stress in *Cxcr4*-cKO mice (Fig. 1r), increased NADPH diaphorase labeling points toward ER stress.

Overall, we found no major changes in olfactory epithelium morphology after *Cxcr4* knockout in horizontal basal cells at steady state, as expected. However, there was higher cell stress. Additionally, we observed a change in the zonal organization of the epithelium. The D-zone, which is characterized by the expression of *Nqo1* and certain olfactory receptors, extended further into the ventral epithelium than in wild-type animals. The next objective was to analyze injury-induced regeneration in *Cxcr4*-cKO mice.

### Absence of CXCR4 Improves the Activation of Horizontal Basal Cells After Injury

**Fig. 2:**
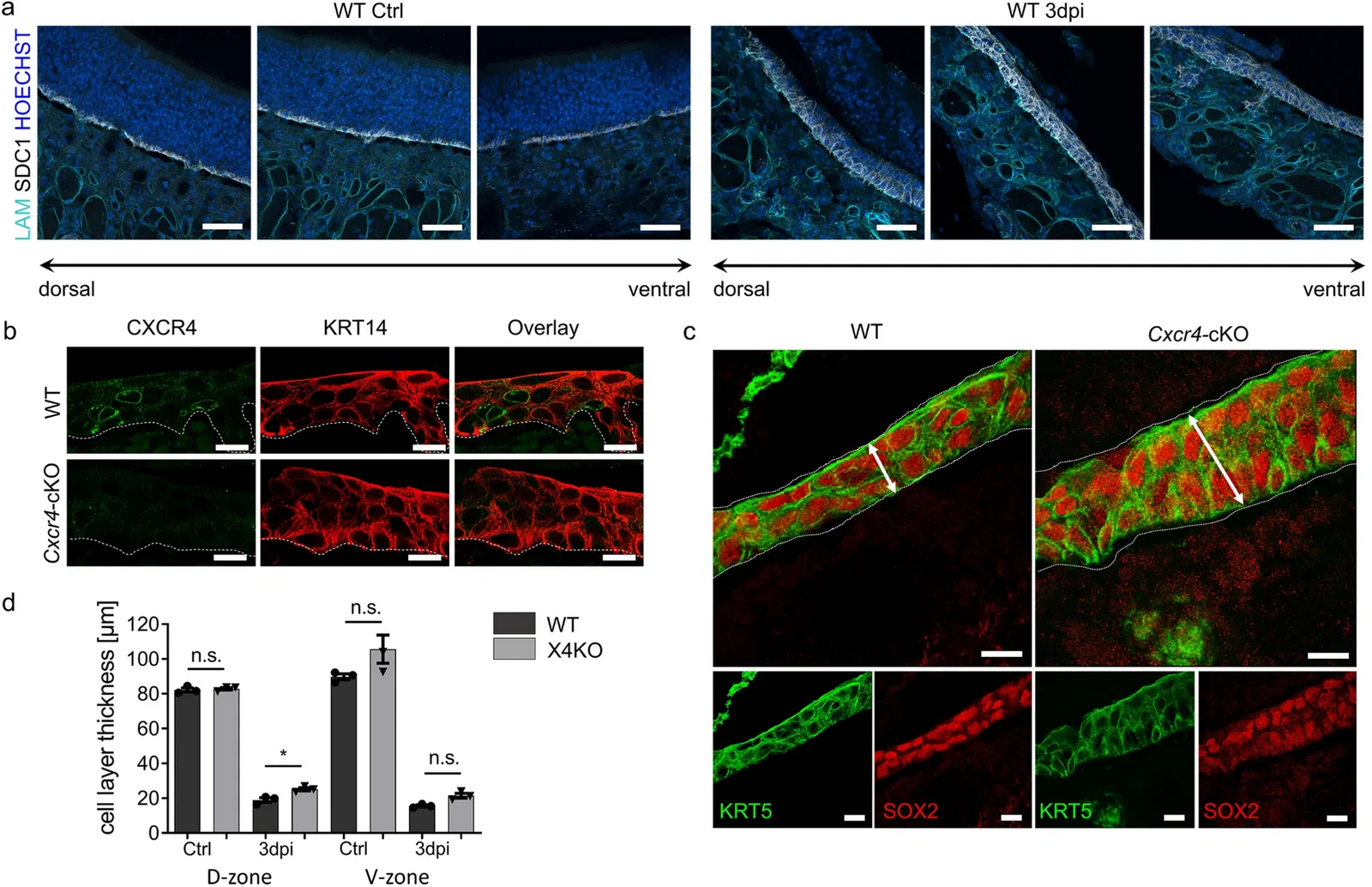
Initial phase of injury-induced regeneration in *Cxcr4*-cKO olfactory epithelium. **a** Confocal images of the olfactory epithelium along the dorsal–ventral axis in WT control animals and WT mice 3 days post-injection (3 dpi) of methimazole, showing most complete detachment of the olfactory epithelium at 3 dpi, apart from activated horizontal basal cells (white, syndecan 1 (SCD1)) and the lamina propria (visible by staining with laminin (LAM)). **b** Olfactory epithelium at 3 dpi, showing activated horizontal basal cells express KRT14 (red) and CXCR4 (green) in WT mice. The epithelium in *Cxcr4*-cKO mice was thicker compared to WT and contained fewer CXCR4-labeled cells. **c** All cells of the epithelium expressed the basal cell markers KRT5 (green) and SOX2 (red) at 3 dpi. Arrows illustrate increased thickness of the epithelium in *Cxcr4*-cKO mice. **d** Quantification of epithelium thickness (control and 3 dpi) in the dorsal (D-zone) and ventral (V-zone) part of the epithelium. Scale bars: **a** 20 µm; **b**, **c** 10 μm. Dotted lines represent the basal lamina and the apical border of the epithelium. Quantification of *n* = 3 animals per group, Student’s *t* test, error bars represent SEM, ^*^p < 0.05

Following injury induced by a single intraperitoneal injection of methimazole, all cells of the olfactory epithelium are destroyed, except the horizontal basal cells. These are activated to regenerate globose basal cells (neuronal stem cells), as well as all other cells of the olfactory epithelium [50]. We therefore used methimazole to study injury-induced neurogenesis in *Cxcr4*-cKO mice. Three days after injection (3 dpi, days post-injection), the olfactory epithelium has almost completely detached, leaving only the horizontal basal cells and the lamina propria (Fig. 2a). In WT mice, CXCR4 expression was not found in quiescent horizontal basal cells [13] but was expressed in activated horizontal basal cells after injury (Fig. 2b). Additionally, activated horizontal basal cells expressed SOX2 (SRY-box transcription factor 2) and expanded (Fig. 2c) but still expressed KRT14 and KRT5, as seen in the quiescent state. In this early phase of activation, the thickness of the olfactory epithelium was increased in *Cxcr4-*cKO compared to WT animals, but difference was only significant in the D-zone of the epithelium (Fig. 2d). Before injury, the thickness of the epithelium was not different between WT and *Cxcr4-*cKO mice (Fig. 2d).

### Improved Injury-Induced Neurogenesis in the Olfactory Epithelium of Cxcr4-cKO Mice

**Fig. 3:**
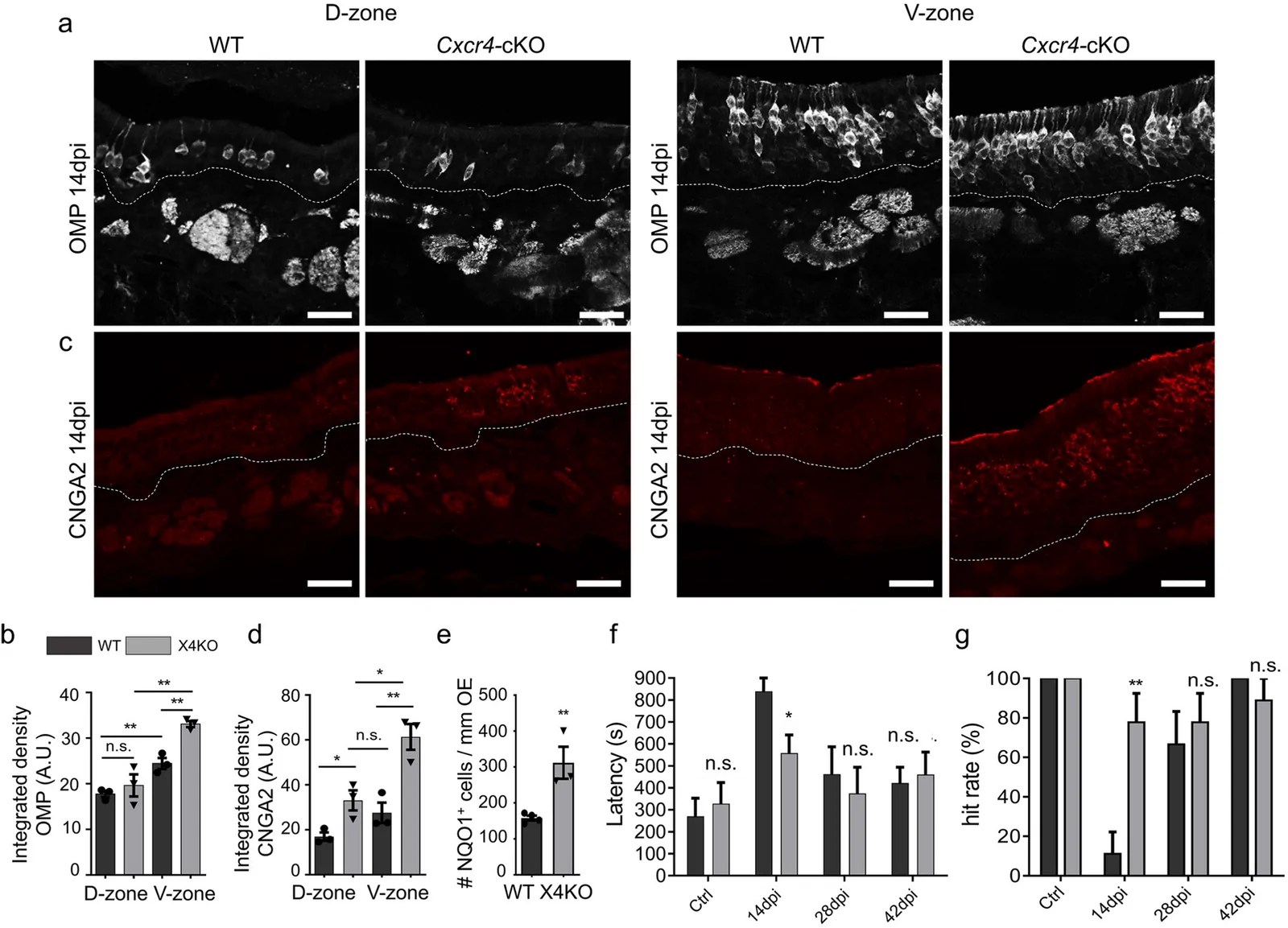
Accelerated regeneration in *Cxcr4*-cKO after injury. **a** OMP-positive neurons in the D- and V-zone of the olfactory epithelium (14 dpi). **c** Quantification of OMP staining intensities, showing increased labeling in the V-zone of the WT and *Cxcr4*-cKO but no significant difference in the D-zone. **c** CNGA2 (cyclic nucleotide-gated channel alpha 2) was localized in neuronal cell somata in the D-zone; ciliary labeling was mostly detectable in the V-zone. **d** Quantification of CNGA2 staining intensities, showing significantly improved regeneration in the *Cxcr4*-cKO. **e** Quantification of D-zone-specific NQO1-positive cells indicated an increase in the *Cxcr4*-cKO. **f** At 14 dpi, WT and *Cxcr4*-cKO mice were slower than noninjured controls in finding a hidden cookie; *Cxcr4*-cKO mice were faster than WT mice. **g** Hit rate after 900-s exploration time. *Cxcr4*-cKO mice were significantly better in cookie finding at 14 dpi. Scale bars: **a**, **c** 20 μm. Dotted lines represent the basal lamina of the epithelium. Quantification of *n* = 3 animals per group (**b**, **d**, **e**), *n* = 9 (**f**, **g**). Student’s *t* test, error bars represent SEM, ^*^p < 0.05, ^**^p < 0.01

Two weeks after injury (14 dpi), elevated neurogenesis was observed throughout the epithelium, albeit with pronounced regional differences. In general, the D-zone contained fewer mature OMP-positive neurons compared to the V-zone (Fig. 3a), consistent with previous findings showing faster maturation of V-zone neurons [51]. In *Cxcr4-*cKO mice, neuronal regeneration was faster compared to WT mice with more OMP-expressing mature neurons in all regions (Fig. 3a, b). Increased ciliary localization of CNGA2 in *Cxcr4-*cKO also indicates faster neuronal regeneration (Fig. 3c, d). Moreover, the number of NQO1-positive cells in the D-zone was increased in *Cxcr4*-cKO mice (Fig. 3e), similar to the homeostatic conditions.

To investigate if these changes are functionally relevant, we analyzed *Cxcr4-*cKO mice in a general olfactory ability assessment test, where we observed mice as they locate a piece of food hidden within bedding material based on olfactory cues, called hidden cookie test [52]. In agreement with the observation that the olfactory epithelium showed increased numbers of OSNs in *Cxcr4-*cKO mice, mice were faster to find the hidden cookie at 14 dpi (Fig. 3f) and showed more success in finding the cookie (Fig. 3g). At later time points, when the epithelial regeneration was more advanced (28 dpi and 42 dpi), the cookie finding performance was similar in WT and *Cxcr4*-cKO mice (Fig. 3f, g).

Taken together, we describe here an enhanced early phase of injury-induced olfactory epithelium regeneration in *Cxcr4*-cKO mice, characterized by the accelerated formation of functional sensory neurons.

### Perturbation Analysis of Regulated Transcription Factors Supports Increased Neuronal Differentiation in Cxcr4-cKO Mice

In order to get a more comprehensive understanding of the effects of CXCR4 on neurogenesis, we conducted a comparative transcriptomic analysis. Visualization of the differential expression (DE) analysis showed that samples of WT and *Cxcr4-*cKO control mice were highly similar (Fig. S3a), as expected. At 14 dpi, we identified 152 genes down and 423 genes upregulated in *Cxcr4-cKO* mice (applying a threshold of FC > 1.5 and *p* adjusted < 0.05, Fig. S3b). We then identified the specific cell types contributing to the changes in gene expression by employing transcriptomic expression-based analysis using published single-cell RNA sequencing data [24]. We found that regulated genes in the early phase of regeneration (14dpi) are mostly expressed in the neuronal lineage (Fig. S3c). As *Cxcr4* is expressed by neuronal precursor cells [13], the absence of *Cxcr4* could explain dysregulated gene expression in the neuronal lineage.

**Fig. 4:**
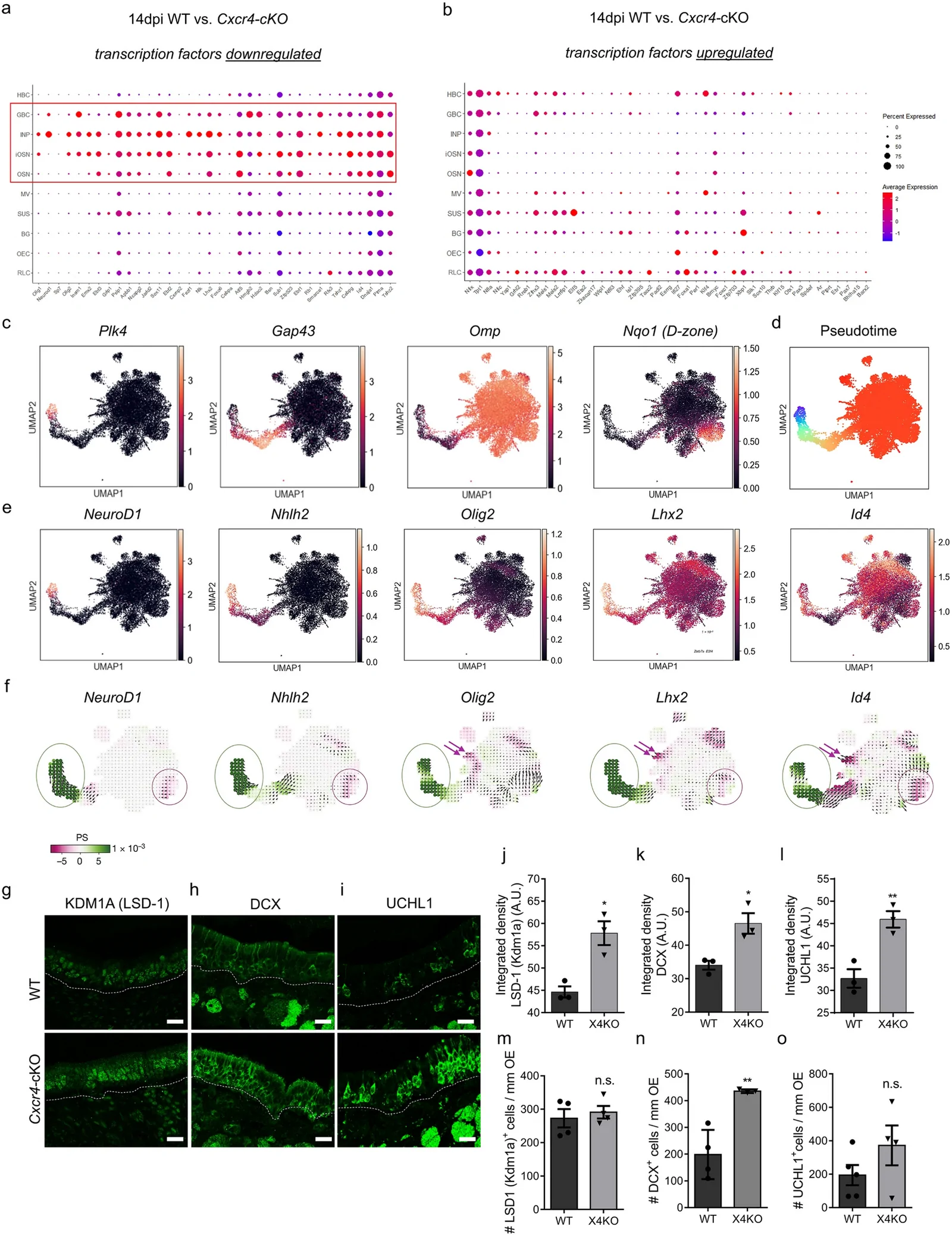
CXCR4 depletion activates transcription factors involved in neurogenesis. **a**,** b** Dot plots (14 dpi) visualizing localization of regulated transcription factors mainly in the neuronal lineage. Dot size represents the relative proportion of cells expressing the gene; color indicates average expression level. HBC: horizontal basal cell; GBC: globose basal cell; INP: immediate neuronal precursor; iOSN: immature olfactory sensory neuron; OSN: olfactory sensory neuron; MV: microvillar cell; SUS: sustentacular cell; RLC: respiratory-like cells. **c** UMAP presentations of the neuronal lineage of the olfactory epithelium: immediate neuronal progenitor cells *Plk4*, immature neurons *Gap43*, mature OSNs *Omp*, D-zone neurons *Nqo1*. **d** Pseudotime analysis showing the developmental trajectory, from blue olfactory progenitors to red mature neurons. **e** UMAP presentations showing localization of regulated transcription factors *Neurod1* (immediate neuronal progenitors), *Nhlh2* and *Olig2* (immediate neuronal progenitor cells and immature neurons), and *Lhx2* and *Id4* throughout the neuronal lineage. **f** In silico transcription factor knockout (CellOracle analysis) of *Neurod1*, *Nhlh2*, *Olig2*, *Lhx2*, and *Id4*, transcription factors that were downregulated in *Cxcr4-*cKO mice. Vector maps show transitions in cell identity; color codes show the directionality of the perturbation vector to the natural differentiation vector. Positive perturbation scores (green) in immature neuronal progenitors/immature neurons (encircled green areas) indicate that reduced expression of these transcription factors promotes neuronal differentiation. Negative perturbation scores (purple) in the D-zone show reduced differentiation into D-zone neurons. *Olig2*, *Lhx2*, and *Id4* simulations show noticeable negative scores for a very small cluster of mature neurons (purple arrowhead). Immunofluorescence labeling of the olfactory epithelium of WT and *Cxcr4*-cKO mice at 14 dpi. **g** KDM1A. **h** DCX. **i** UCHL1. Quantification of immunofluorescence intensities (**j**,** k**,** l**) and cell numbers (**m**,** n**,** o**) of KDM1A, DCX, and UCHL1 in WT and *Cxcr4*-cKO mice (*n* = 3–5 animals per group, Student’s *t* test, error bars represent SEM, ^*^p < 0.05, ^**^p < 0.01). Scale bars 10 µm. Dotted lines represent the basal lamina

Furthermore, we found that most of the differentially expressed transcription factors were also expressed by cells of the neuronal lineage (Fig. 4a, b). To analyze the effects of the regulated transcription factors on cell identities, we used CellOracle analysis to perform in silico transcription factor perturbations. CellOracle is a machine learning–based approach to model changes in phenotypes as a result of transcription factor perturbation [27]. UMAP (uniform manifold approximation and projection) presentations were used to show the position of cell types of the olfactory epithelium throughout neurogenesis (Fig. 4c). Immediate neuronal progenitor cells express *Plk4* (serine/threonine-protein kinase 4), immature neurons *Gap43*, and mature OSNs *Omp*. In addition, the position of the D-zone neurons in the map is revealed by *Nqo1* expression (Fig. 4c). Pseudotime analysis shows the developmental trajectory, from olfactory progenitors (blue) to mature neurons (red) (Fig. 4d). UMAPs of transcription factors that were downregulated in *Cxcr4-cKO* mice showed *Neurod1* in immediate neuronal progenitor cells, *Nhlh2 *(nescient helix loop helix 2) and *Olig2* (oligodendrocyte transcription factor 2) in immediate neuronal progenitor cells and immature neurons, and *Lhx2 *(Lim homeobox 2) and *Id4* (inhibitor of DNA binding 4) throughout the neuronal lineage (Fig. 4e). We then employed in silico perturbation to simulate downstream shifts in gene expression following knockout of these transcription factors and visualized the respective results as vector maps (Fig. 4f). In all cases analyzed, we found positive perturbation scores (green) in immediate neuronal progenitors/immature neurons (Fig. 4f, encircled green), indicating that reduced expression of the transcription factors promotes neuronal differentiation. This is in perfect agreement with the observed increased number of neurons and improved cookie smelling of *Cxcr4-cKO* animals at 14 dpi (Fig. 3). In *Neurod1*, *Nhlh2*, *Lhx2*, and *Id4* simulations, negative perturbation scores were detected in a subpopulation of neurons expressing *Nqo1* (Fig. 4f, encircled purple). Accordingly, the knockout is predicted to delay or block differentiation of *Nqo1*-positive neurons located in the dorsomedial part of the epithelium. Moreover, we detected noticeable negative scores for a very small cluster of mature neurons in case of *Olig2*, *Lhx2*, and *Id4* in silico knockout results (Fig. 4f, purple arrowheads). Interestingly, genes contributing to this cluster were odorant binding proteins and lipocalins (Fig. S3d, e) and were among the most markedly regulated genes in the transcriptomic analysis. A distinct cluster of mature vomeronasal neurons also expressing odorant binding proteins and lipocalins, usually enriched in nonneuronal supporting or secretory gland cells, has been described as putative secretory vomeronasal sensory neurons [53].

The increased abundance of immature neurons was also detectable by immunohistochemistry. *Cxcr4-*cKO mice showed increased staining of KDM1A (lysine (K)-specific demethylase 1 A, LSD1) (Fig. 4g, j), which is highly expressed in dividing globose basal cells and declines around the time when OSNs mature [54]. However, cell number quantification of KDM1A-positive cells did not reach significance (Fig. 4m). Other markers for immature neurons such as DCX (Fig. 4h, k, n) and TUBB3 (tubulin beta 3) (Fig. S3f) were also increased. Similar cell numbers but enhanced staining intensities were also observed for UCHL1 (ubiquitin C-terminal hydrolase L1, also called PGP9.5) (Fig. 4i, l, o), which is a ubiquitous neuronal protein expressed in the late stages of maturation. In addition, increased staining intensities of synapsin-1 (SYN1, Fig. S3g) and GM130 (Golgi matrix protein of 130 kDa, Fig. S3h, i) showed that olfactory neurons in *Cxcr4*-cKO mice underwent faster maturation.

Taken together, perturbation analysis of the regulated transcription factors and the increased abundance of immature neurons and mature neuronal cells in the olfactory epithelium of *Cxcr4-*cKO mice confirmed improved differentiation or survival of neuronal cells.

### Axonal Outgrowth Is Increased in Cxcr4-cKO Mice

**Fig. 5:**
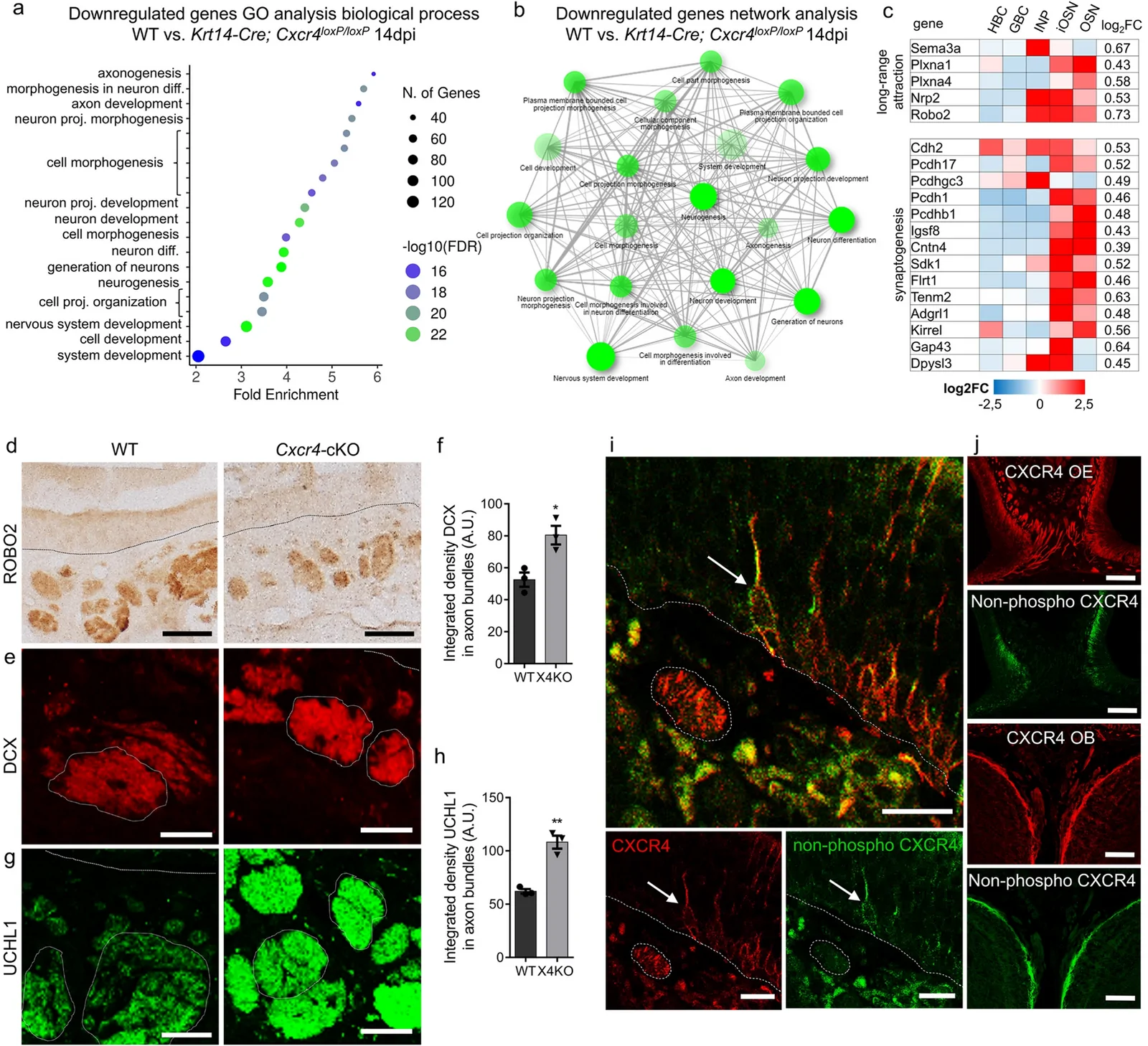
CXCR4 depletion affects axon extension. **a** Dot plot visualization of GO term enrichment for biological processes for the downregulated genes from *Cxcr4*-cKO olfactory epithelium identified exclusively neuron morphogenesis-related pathways, highlighted based on gene count (dot size), fold enrichment (position), and significance (color) as determined by adjusted *p* values in the GO analysis. Similar GO terms are summarized for simplification. **b** Network analysis of GO term enrichment for biological processes showing tight clustering of the affected processes. **c** Axon guidance molecules show altered expression in *Cxcr4*-cKO mice (HBC horizontal basal cell, GBC globose basal cell, INP immediate neuronal progenitor cell, iOSN immature olfactory neurons, OSN mature olfactory neuron). **d** Immunohistochemical staining of ROBO2 (roundabout homolog 2) in the D- and V-zone of the olfactory epithelium at 14 dpi showing reduced labeling in *Cxcr4*-cKO mice. D-zone of the olfactory epithelium of WT and *Cxcr4*-cKO mice at 14 dpi; immunofluorescent labeling of DCX (**e**) and UCHL1 (**g**) in axon bundles in the lamina propria. Quantification of immunofluorescent staining of DCX (**f**) and UCHL1 (**h**) in lamina propria axon bundles of WT and *Cxcr4*-cKO mice, showing increased labeling in *Cxcr4*-cKO mice. **i** Basal olfactory epithelium and the lamina propria. Immature OSNs express CXCR4 (red). CXCR4 activation is shown by poor labeling with UMB-2 antibody (green), which stains only nonphosphorylated CXCR4. CXC4 is activated in most but not all neurons (arrow pointing to neuron with co-labeling of CXCR4 and UMB-2). Axon bundles (encircled area) are not labeled by UMB-2 (green), showing CXCR4 activation in the lamina propria. **j** Images of the olfactory system showing immunolabeling of CXCR4 (red) and nonactivated CXCR4 (UMB-2, green). CXCR4 is present on axon bundles in the lamina propria and in the outer nerve layer surrounding the olfactory bulb. Low intensity of UMB-2 labeling in axon bundles shows marked activation of the receptor (*n* = 3 animals per group, Student’s *t* test, error bars represent SEM, ^*^p < 0.05, ^**^p < 0.01). Scale bars: 50 µm (**d**), 20 µm (**e**, **g**, **i**), 100 µm (**j**). Dotted lines represent the basal lamina or axon bundles

Next, we performed Gene Ontology (GO) analysis of genes regulated in *Cxcr4*-cKO mice at 14 dpi. To improve the power to detect significant pathways, we separated the up- and downregulated genes in the enrichment analysis, since genes in a pathway that is disturbed tend to be up- or downregulated similarly, owing to their functional links [55]. The top 20 GO terms of the downregulated genes within the biological process category, filtered by significance using *p*-adjusted values, were exclusively neurogenesis-related terms, the top terms being linked to axonogenesis (Fig. 5a). Network analysis of the GO terms revealed tight clustering, showing that neurogenesis represents the most relevant affected process in *Cxcr4-*cKO olfactory epithelium (Fig. 5b). Analysis of the transcriptomic data moreover revealed differential expression of well-known axon guidance genes in *Cxcr4*-cKO mice (Fig. 5c). Decreased levels of ROBO2 (roundabout guidance receptor 2), a protein that is enriched in the D-zone [56], were confirmed in the axon bundles of the lamina propria (Fig. 5d). Analysis of ubiquitous marker proteins for olfactory neurons (DCX and UCHL1) in axon bundles of the lamina propria revealed increased levels in *Cxcr4*-cKO mice (Fig. 5e–h). Together, these findings suggest that Cxcr4 deletion alters axonal development within the lamina propria, with reduced expression of axon guidance-associated ROBO2 and increased abundance of immature axonal markers.

Olfactory axons projecting through the lamina propria are positive for CXCR4 (Fig. 5i). As expected from the marked expression of the CXCR4 ligand CXCL12 in the lamina propria [14], CXCR4 seems to be fully activated in the axon bundles of the lamina propria. Nonactivated and thereby nonphosphorylated CXCR4 was specifically labeled with the anti-CXCR4 antibody UMB-2, which recognizes the C-terminal CXCR4 epitope only when the 346SSS348 cluster is not phosphorylated [57]. Absence of UMB-2 labeling therefore shows activation of CXCR4 in the axons growing through the lamina propria (Fig. 5i, j). Combined, we show that CXCR4 deficiency promotes axon extension in lamina propria during early injury-induced neurogenesis.

### Disturbed Axonal Projection in Cxcr4-cKO Mice

**Fig. 6:**
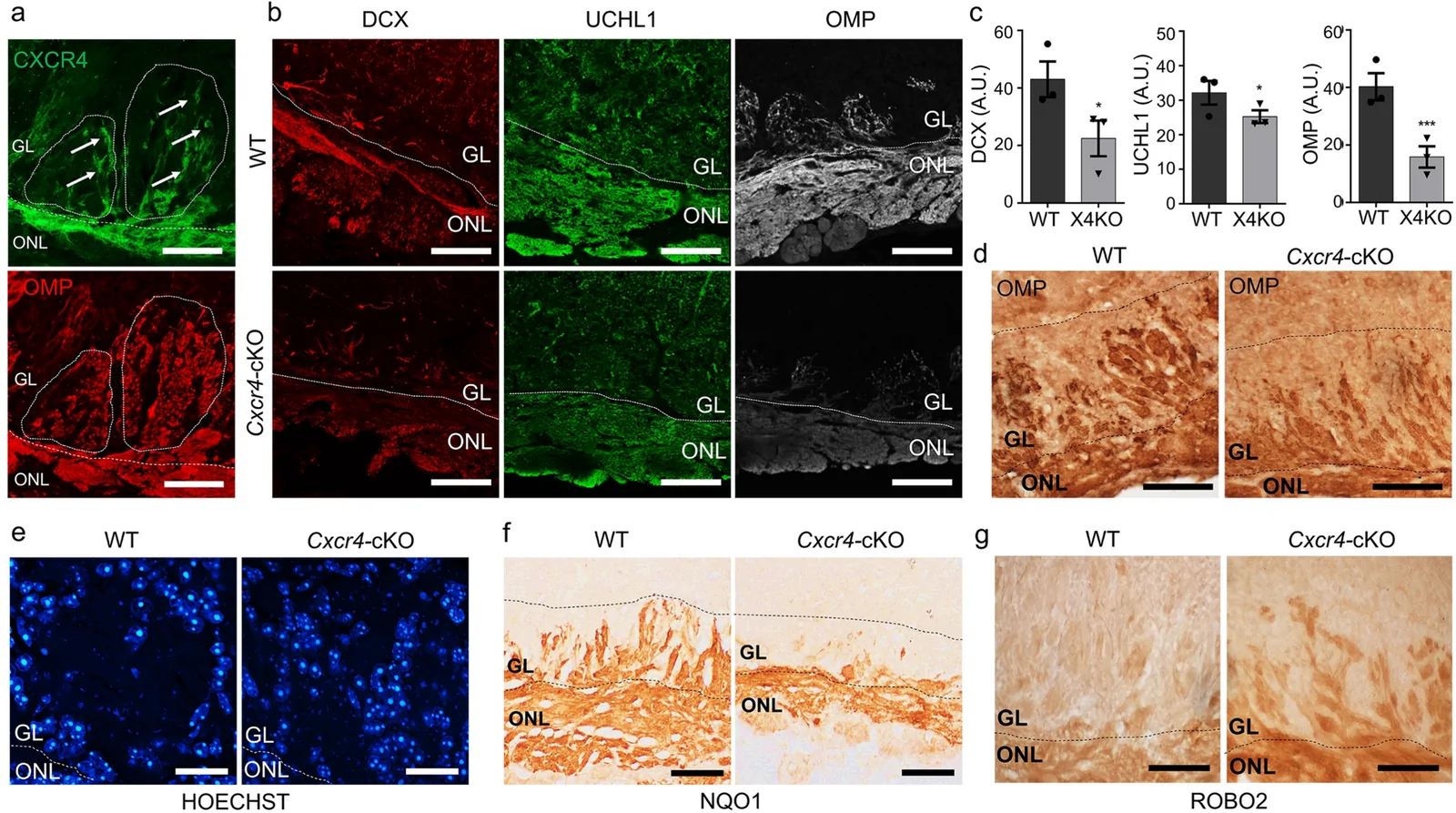
Reduced CXCR4 expression impairs axon targeting. **a** CXCR4 (green) was present in the outer nerve layer surrounding the olfactory bulb; the glomeruli showed only sparse immunostaining (white arrows). Border of the glomerulus (encircled area) was identified by OMP-labeling (red). **b** Confocal images of coronal sections through the olfactory bulb showing the outer nerve layer at the medial site of the bulb of WT and *Cxcr4*-cKO mice at 14 dpi; immunofluorescence shows labeling of DCX, UCHL1, and OMP. The glomerular layer showed only sparse labeling in *Cxcr4*-cKO. **c** Quantification of immunofluorescent staining intensities in the outer nerve layer showing lower levels of DCX, UCHL1, and OMP in *Cxcr4*-cKO mice (14 dpi). **d** Immunohistochemical DAB staining of OMP at 42 dpi in the olfactory bulb showing not only decreased OMP-labeling in *Cxcr4*-cKO mice but also disturbed glomerular refinement. **e** Staining of nuclei with Hoechst in the olfactory bulb displayed altered structures of the glomeruli. **f** Immunohistochemical DAB staining for NQO1 indicating obviously reduced staining in the glomerular layer of the olfactory bulb after regeneration in *Cxcr4*-cKO mice. **g** Immunohistochemical DAB staining of ROBO2 at 42 dpi showed strong labeling of ROBO2 within the glomeruli in the *Cxcr4*-cKO compared to WT. For quantification, *n* = 3 animals per group were used (Student’s *t* test, error bars represent SEM, ^*^p < 0.05, ^**^p < 0.01, ^***^p < 0.001). Scale bars 50 µm (**a**, **b**, **d**, **f**, **g**), 20 µm (**e**). ONL: outer nerve layer, GL: glomerular layer. Dotted lines represent the separation between GL and ONL or glomeruli

Axons of olfactory neurons grow through the lamina propria and finally project to the olfactory bulb. In the outer nerve layer of the olfactory bulb, OSN axons defasciculate before synapsing with mitral cells in the glomeruli. These glomeruli showed only sparse CXCR4 labeling with only few CXCR4-positive axons in WT mice (Fig. 6a), consistent with rapid decline of CXCR4 expression during neuronal maturation [13] and with rapid internalization of the receptor at high ligand concentrations.

There is a strong correlation between the location of the olfactory neurons within the epithelium and axon targeting in the olfactory bulb. Neurons of the D-zone project to the dorsal region of the olfactory bulb, whereas V-zone neurons innervate the ventral region [58]. Despite the fact that axonal outgrowth in the lamina propria of *Cxcr4*-cKO mice was increased at 14 dpi (Fig. 5), the dorsal olfactory bulb displayed reduced staining of DCX, UCHL1, and OMP (Fig. 6b, c) may be due to the observed altered expression of adhesion genes. Additionally, after injury-induced neurogenesis was nearly completed in the olfactory epithelium (42 dpi), *Cxcr4*-cKO mice lacked clear glomerular structures in the dorsal region of the olfactory bulb (Fig. 6d). Glomeruli often displayed wider, more diffuse borders, and nuclear staining failed to reveal the typical circular arrangement of periglomerular cells (Fig. 6e). Axon terminals that were positive for NQO1, a marker for the D-zone neurons of the olfactory epithelium, were absent from the dorsal olfactory bulb (Fig. 6f). In addition, the axon targeting protein ROBO2, which is also expressed in a high dorsomedial to low ventrolateral gradient [56], was barely detectable in glomeruli of the WT olfactory bulb and was mislocalized and clearly detected in glomeruli of *Cxcr4*-cKO mice (Fig. 6g). Therefore, the deletion of *Cxcr4* promotes the growth of axons in the lamina propria during regeneration. However, it impairs the subsequent targeting of axons and the organization of glomeruli in the dorsal olfactory bulb.

### Loss of CXCR4 Impairs Long-Term Regeneration and Maintenance of D-Zone Olfactory Neurons

**Fig. 7:**
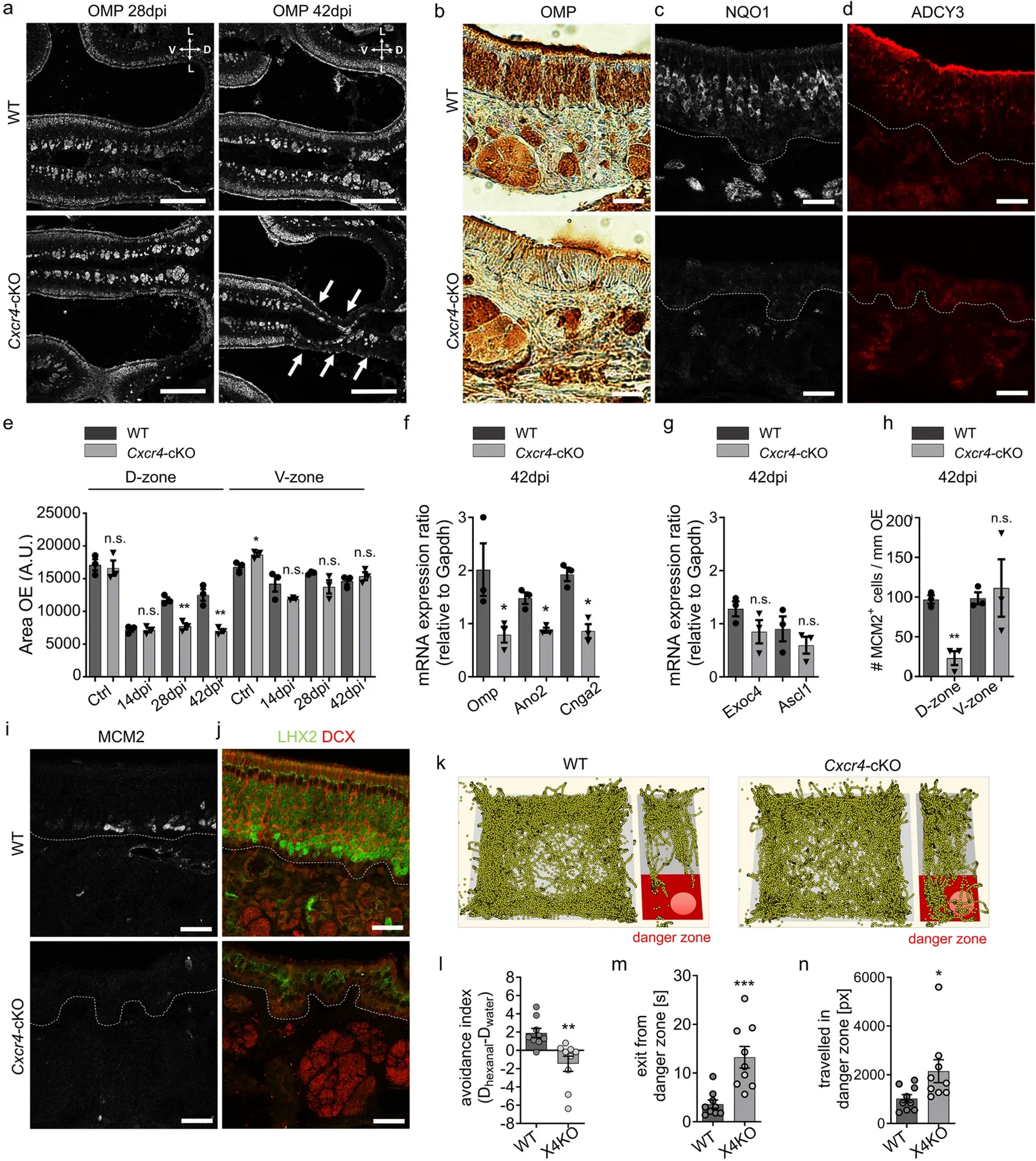
CXCR4 depletion leads to loss of D-zone neurons. **a** Immunofluorescence labeling of OMP. At 28 dpi, OMP labeling appeared similar in WT and *Cxcr4*-cKO mice; at 42 dpi, the D-zone showed signs of degeneration in *Cxcr4*-cKO mice. L = lateral; V = ventral, D = dorsal, arrows point to degenerated parts. **b** Quantification of caspase 3 labeling in the D- and V-zone at 14, 28, and 42 dpi showing more dying cells at 14 dpi in *Cxcr4*-cKO. **b** 3,3-Diaminobenzidine labeling of OMP-positive neurons in the D-zone at 42 dpi. Immunofluorescent staining for NQO1-positive (**c**) or ADCY3-positive (**d**) mature neurons in the D-zone of the epithelium; both proteins were absent in the D-zone of *Cxcr4*-cKO (42 dpi). **e** Quantification of the total thickness of the olfactory epithelium in both zones, indicating a difference in the D-zone of in *Cxcr4*-cKO mice at 28 and 42 dpi. Quantitative PCR from WT and *Cxcr4*-cKO animals (42 dpi). Expression of mature neuron markers *Omp*, *Ano2*, and *Cnga2* was reduced in *Cxcr4*-cKO (**f**); globose basal cell markers *Exoc4* and *Ascl1* were similar between WT and *Cxcr4*-cKO (**g**). **h** Quantification of MCM2-positive globose basal cells at 42 dpi, showing significant reduction of proliferating cells in the D-zone of in *Cxcr4*-cKO mice. **i** Immunofluorescent labeling of MCM2 in the D-zone and V-zone of WT and *Cxcr4*-cKO mice. **j** Immunofluorescent labeling of LHX2 (LIM homeobox 2, green) and DCX (red) showing that neuronal precursors and immature neurons are almost absent in the D-zone of *Cxcr4*-cKO mice at 42 dpi. **k** Tracks of mice moving around in the cage (3 min). WT mice avoid the hexanal source; *Cxcr4*-cKO mice enter the area close to the hexanal source (red, danger zone) more frequently at 42 dpi. **l** Avoidance index calculated by comparing the distance of mice from the hexanal source with the water control. WT mice avoid hexanal; *Cxcr4*-cKO mice do not. **m** WT mice were much faster to leave the danger zone than *Cxcr4*-cKO mice at 42 dpi. **n** Quantification of the distance (in pixels) traveled showing that *Cxcr4*-cKO mice stayed longer in the danger zone. **a**, **f**, **g**, **h** *n* = 3 animals per group; **k**, **l**, **m** *n* = 9 animals per group; Student’s *t* test, error bars represent SEM, ^*^p < 0.05, ^**^p < 0.01, ^***^p < 0.001. Scale bars 20 µm (**b**, **c**, **d**, **i**, **j**), 100 µm (**a**). Dotted lines represent basal lamina

Mistargeted olfactory axons that fail to make functional synaptic connections have been shown to cause cell death over a period of up to 10 weeks [59]. Four weeks after injury (28 dpi), the olfactory epithelium of *Cxcr4-*cKO mice appeared similar to the WT epithelium. However, we observed an absence of OMP labeling in the D-zone of *Cxcr4*-cKO mice during the subsequent regeneration process (Fig. 7a, arrows). By 42 dpi, the epithelium had almost completely regenerated, containing several layers of sensory neurons. However, OMP expression continued to increase until 92 dpi (Fig. S4).

Higher magnification of the D-zone confirmed absence of OMP-positive OSNs and showed a pseudostratified columnar epithelium (Fig. 7b). NQO1-positive D-zone neurons were nearly absent (Fig. 7c), and ciliary localization of adenylate cyclase type 3 (ADCY3) was missing (Fig. 7d). Measurement of the total thickness of the epithelium revealed a reduced thickness in the D-zone, while other zones were not affected (Fig. 7e). In accordance with the loss of OSNs, expression levels of mature neuronal genes *Omp*, *Ano2*, and *Cnga2* were lower in *Cxcr4-*cKO animals (Fig. 7f). Overall mRNA expression levels of *Exoc4* and *Ascl1*, markers of globose stem cells which regenerate the neuronal lineage, were reduced but showed no significant differences (Fig. 7g). However, cell proliferation (Fig. 7h, i) was markedly reduced in the D-zone of *Cxcr4*-cKO mice, indicating absence of proliferating globose basal cells in the D-zone. In addition, staining of immature neurons with LHX2 and DCX (Fig. 7j) was barely detectable in the epithelium.

The D-zone contains specific populations of olfactory sensory neurons involved in the detection of aversive odorants. WT mice show avoidance behaviors toward predator odorants and spoiled smells, and mice lacking D-zone neurons spend much longer time investigating otherwise aversive acids, aldehydes, amines, ketones, and predator odors, among them hexanal [60]. We therefore tested if *Cxcr4-*cKO mice were able to smell hexanal as aversive odorant after degeneration of the D-zone neurons. 42 dpi WT mice clearly avoided the areas of a test cage close to a source of hexanal, while the *Cxcr4-*cKO mice did not do so (Fig. 7k and Movies 1–3). Analysis of the mean displacement of the mice from the hexanal source compared to a water sample showed that WT mice, but not *Cxcr4*-cKO mice, avoided hexanal (Fig. 7l). *Cxcr4-*cKO mice spent more time exploring the hexanal area (Fig. 7m) and moved around more in the hexanal area (Fig. 7n), demonstrating impaired avoidance behavior.

Together, these results show that after initial fast onset of neurogenesis, knockout of *Cxcr4* ultimately leads to loss of neurons in the D-zone of the olfactory epithelium.

### Cxcr4-cKO Causes Endoplasmic Reticulum (ER) Stress

**Fig. 8:**
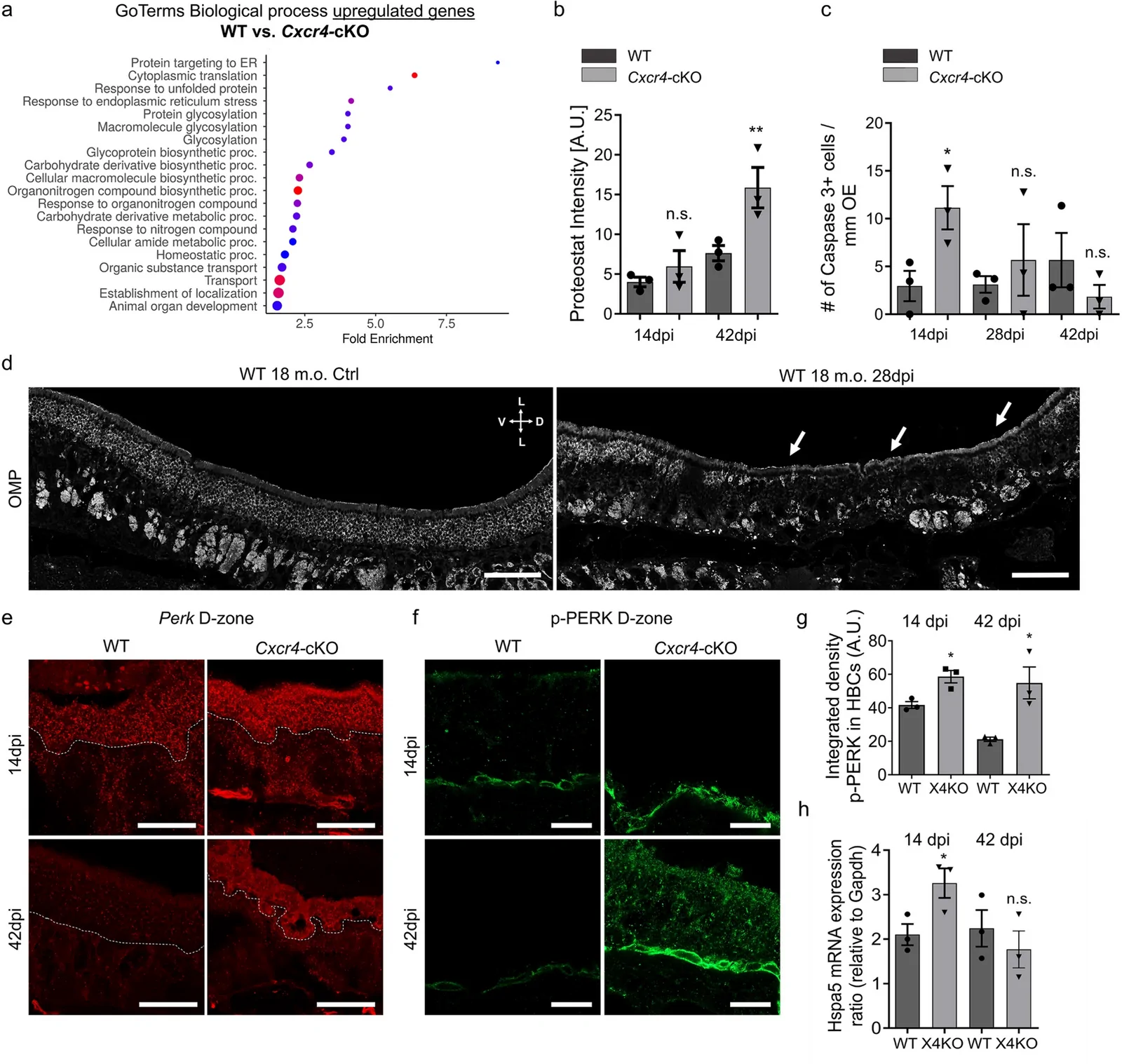
Loss of CXCR4 is associated with ER stress. **a** Dot plot visualization of GO term enrichment for biological processes for the upregulated genes from *Cxcr4*-cKO olfactory epithelium at 42 dpi. Gene counts are represented by dot size, fold enrichment by *x*-axis position, and significance as determined by adjusted *p* values in the GO analysis by color. **b** Quantification of labeling with Proteostat® Aggresome Kit at 14 dpi and 42 dpi. **c** Quantification of caspase 3-labeled cells in the olfactory epithelium at 14, 28, and 42 dpi, showing a significant increase of dying cells at 14dpi in *Cxcr4*-cKO mice. **d** Immunofluorescent labeling of OMP in 18M old control WT mice and at 28 dpi after methimazole treatment. After injury, the epithelium regenerated, except parts of the D-zone. **e** In situ hybridization for *Perk* showing higher expression in the neuronal layer of the olfactory epithelium in *Cxcr4*-cKO mice at 14 dpi and 42 dpi. **f** Immunofluorescent staining of phosphorylated PERK at 14 dpi and 42 dpi, indicating an increase of p-PERK in the horizontal basal cells of *Cxcr4*-cKO mice. **g** Quantification of p-PERK staining intensities in horizontal basal cells (HBCs) showing an increase in *Cxcr4*-cKO mice. **h** Quantitative PCR from olfactory mucosa mRNA at 14 dpi and 42 dpi. The ER stress marker Hspa5 was increased at 14 dpi in *Cxcr4*-cKO animals. **b**, **c**, **g**, **h** *n* = 3 animals per group; Student’s *t* test, error bars represent SEM, ^*^p < 0.05, ^**^p < 0.01. Scale bars 50 µm (**d**, **e**), 10 µm (**f**)

The finding that knocking out *Cxcr4* caused loss of neurons in the D-zone of the olfactory epithelium some weeks after injury led us to investigate possible explanations. Specific GO term analysis of upregulated genes in *Cxcr4*-cKO mice identified genes involved in “protein targeting to the ER (endoplasmic reticulum),” “cytoplasmic translation,” and “responses to unfolded proteins” (Fig. 8a). The ER stress marker Proteostat® confirmed the increased abundance of misfolded proteins in *Cxcr4*-cKO mice and their accumulation over time (Fig. 8b). Moreover, caspase 3 labeling showed more apoptotic neurons in the D-zone at the beginning of neurogenesis and axon outgrowth (14 dpi, Fig. 8c). Increased apoptosis and the activation of ER stress–related pathways contribute to age-related pathologies. An analysis of injury-induced regeneration in aged WT animals (18 m.o.) in fact showed that neurons in the D-zone did not regenerate, despite regeneration of other segments of the olfactory epithelium (Fig. 8d).

PERK (protein kinase RNA-like ER kinase, gene name EIF2AK3 (eukaryotic translation initiation factor 2 alpha kinase 3)) is a key sensor protein in the ER which is activated during ER stress, when unfolded proteins build up. In situ hybridization of *Perk* mRNA revealed increased expression throughout the olfactory epithelium in *Cxcr4*-cKO mice, possibly as a result of elevated cell stress (Fig. 8e). In the canonical PERK pathway, sensing of misfolded proteins induces PERK auto-phosphorylation, and phosphorylated PERK subsequently phosphorylates eukaryotic translation initiation factor 2 alpha (eif2α) to attenuate global protein translation and to upregulate chaperone expression. There was a higher level of phosphorylated PERK in horizontal basal cells of WT mice during ongoing regeneration (14 dpi) compared to the time point when neurogenesis has largely been completed (Fig. 8f, g), suggesting ER stress during activation. PERK phosphorylation in the D-zone of *Cxcr4*-cKO mice was more pronounced, consistent with upregulation of ER stress–related proteins. Moreover, PERK phosphorylation in the D-zone of *Cxcr4*-cKO mice did not decline over time (42 dpi, Fig. 8f, g). Furthermore, PERK phosphorylation could lead to increased expression of *Hspa5* (heat shock protein family member 5), an ER chaperone (Fig. 8h).

It has been shown that ER stress regulates expression of axon guidance and cell adhesion genes in olfactory neurons [4]. A number of genes associated with axon guidance found to be regulated in *Cxcr4*-cKO mice (PLXNA4, NRP2, ROBO2, PCDH17, CNTN4, TENM2, and GAP43, shown in Fig. 5c) are also known to be regulated by ER stress [4]. Hence, the increased stress response in *Cxcr4*-cKO animals might also cause the loss of D-zone neurons as well as altered glomerular targeting.

### Basal Cells of the Metaplastic Epithelium are Different from Horizontal Basal Cells

**Fig. 9:**
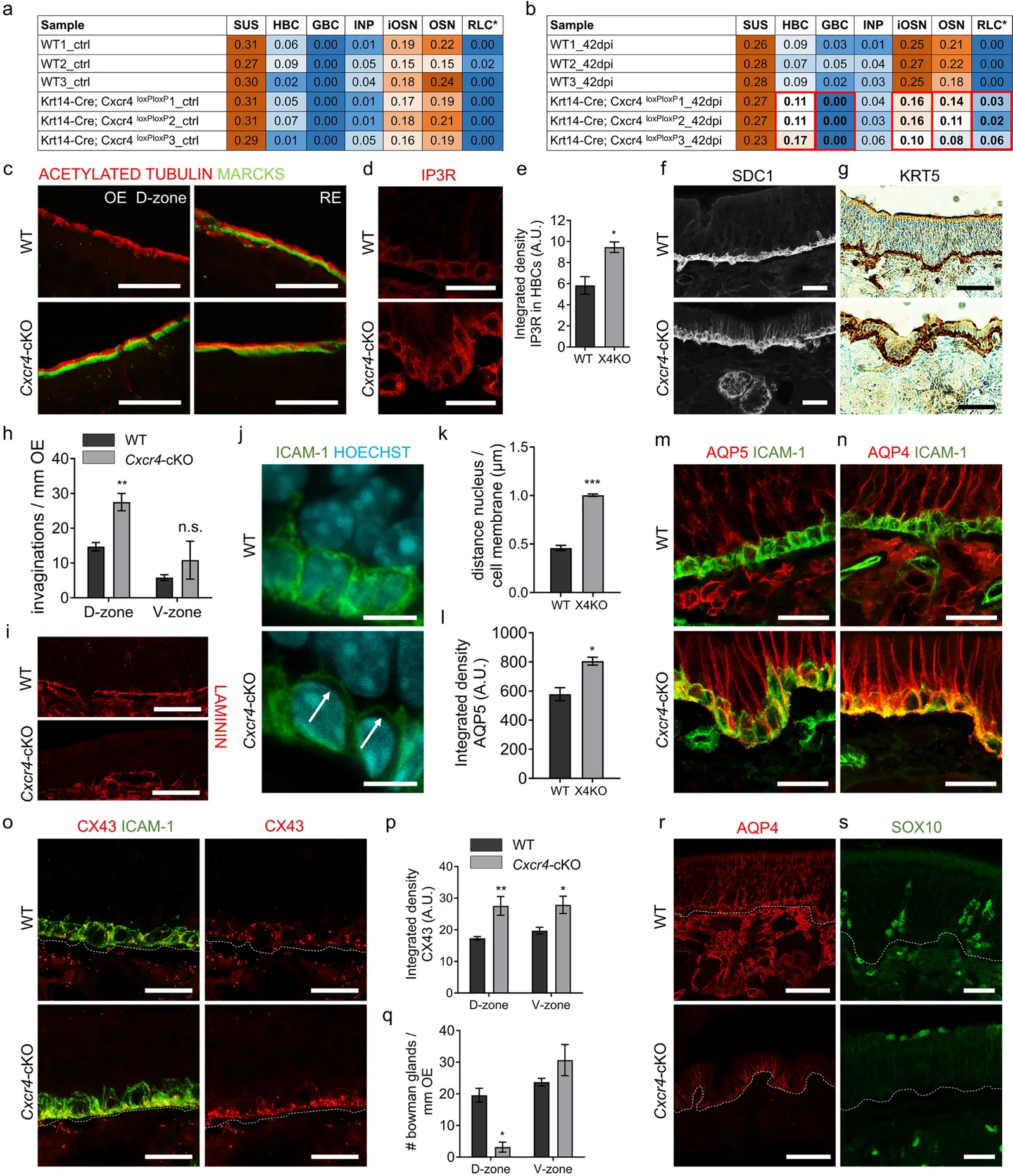
Basal cells of the metaplastic D-zone are different from horizontal basal cells of the olfactory epithelium. **a**,** b** Cell type deconvolution from bulk RNA-seq data via CIBERSORTx, shown as relative fractions normalized to 1 across all cell subsets. Significant differences are highlighted by bold letters. **a** No difference in the relative fractions of cell types between WT and *Cxcr4*-cKO mice at steady state. **b** Cell type deconvolution of 42 dpi data shows reduced numbers of OSNs and increased numbers of respiratory-like cells in *Cxcr4*-cKO compared to WT mice. **c** Labeling of acetylated tubulin (red) and MARCKS (green) in the olfactory epithelium (D-zone) and in the respiratory epithelium (RE) of WT and *Cxcr4*-cKO mice. **d** Immunofluorescent labeling of IP3R3 indicates increased activation of basal cells in *Cxcr4*-cKO mice at 42 dpi. **e** Quantification of IP3R3 staining intensities in basal cells showing a significant increase in *Cxcr4*-cKO mice. **f** Immunofluorescent labeling of SCD1 in horizontal basal cells was increased in *Cxcr4*-cKO animals. **g** Immunohistochemical DAB staining for KRT5 also indicates stronger labeling of basal cells in *Cxcr4*-cKO mice. Furthermore, *Cxcr4*-cKO mice show marked invaginations of the basal lamina, which were not found in WT mice. **h** Quantification of basal lamina invaginations. **i** Immunofluorescent staining of laminin indicates reduced labeling of the basal lamina in *Cxcr4*-cKO mice. **j** Immunofluorescent labeling of horizontal basal cells with ICAM1 (green) and HOECHST (blue), showing a rounded cell morphology in *Cxcr4*-cKO mice. **k** Quantification of the distance between the nucleus and the cell membrane (labeled by ICAM1). The distance was increased in *Cxcr4*-cKO mice, demonstrating the difference in cell morphology. **l** Quantification of AQP5 staining intensities in horizontal basal cells. Increased immunofluorescent staining of AQP5 (**m**) or AQP4 (**n**) (red) in *Cxcr4*-cKO mice, basal cells are stained with ICAM1 (green). **o** Immunofluorescent labeling of CX43 (red) as a marker for gap junctions together with ICAM1 indicates increased cell–cell contacts between basal cells of *Cxcr4*-cKO mice. **p** Quantification of CX43 in horizontal basal cells. **q** Quantification of Bowman glands (stained with AQP5) in the olfactory mucosa showing a significant reduction of Bowman glands in the lamina propria of the D-zone of *Cxcr4*-cKO mice. Immunofluorescent staining for AQP4 (**r**) and SOX10 (**s**) also shows the absence of Bowman glands in *Cxcr4*-cKO mice. **e**, **h**, **k**, **l**, **p**, **q** *n* = 3 animals per group; Student’s *t* test, error bars represent SEM, ^*^p < 0.05, ^**^p < 0.01. Scale bars 5 µm (**j**), 10 µm (**c**, **d**, **e**, **m**, **n**, **o**), 20 µm (**r**, **s**)

To gain a more comprehensive understanding of the degeneration of the D-zone in *Cxcr4*-cKO mice, we used CIBERSORTx [33] to estimate the abundances of cell types in the olfactory epithelium after loss of neurons in the D-zone. A published scRNA-seq dataset [24] was used to generate the deconvolution signature matrix, needed to enumerate cell fractions from our transcriptome analysis at 42 dpi. Under homeostatic conditions, we found no alteration in the cellular composition of both genotypes (Fig. 9a). Calculated cell numbers revealed decreased numbers of neurons in *Cxcr4-cKO* mice at 42 dpi (Fig. 9b), demonstrating that the RNA-seq dataset represents the observed phenotype. The analysis also revealed an increased abundance of cells initially classified as “ependymal cells” [24] (here renamed RLC (respiratory-like cells), Fig. 9b), possibly representing the cells in the aneuronal D-zone. Classification as respiratory-like cells was based on analysis of the expression of respiratory epithelium marker genes in the reference dataset. Respiratory epithelium-specific genes were derived from single-cell RNA-seq data of ciliated airway epithelial cells of the human lung [61] and from the Monarch Initiative [62] (Fig. S5a, b). Both datasets showed that the cell cluster classified as “ependymal cells” displays marked similarities to ciliated respiratory cells. Staining with the respiratory epithelium marker MARCKS (myristoylated alanine-rich C-kinase substrate) and acetylated tubulin confirmed that the metaplastic D-zone cells were ciliated and bore a marked resemblance to respiratory epithelial cells (Fig. 9c).

IP3-mediated calcium signaling occurs in activated horizontal basal cells and correlates with the appearance of respiratory barrier cells instead of sensory neurons [63]. Increased levels of IP3R3 (inositol-1,4,5-trisphosphate-receptor, type 3, ITPR3) in basal cells in the metaplastic zone therefore indicates activation (Fig. 9d, e). The basal cells of the metaplastic D-zone expressed markers for horizontal basal cells such as SCD1 (syndecan 1) and KRT5 but at higher levels (Fig. 9f, g). Moreover, we found that the basal lamina, which always appears as straight line in WT mice, showed multiple invaginations (Fig. 9g, h). Moreover, we found reduced levels of laminin at the basal lamina in *Cxcr4*-cKO mice (Fig. 9i). Analysis of published scRNA-seq data [24] revealed that horizontal basal cells express laminins, components of the basal lamina (Fig. S5c). Together, differences of basal cells in the metaplastic epithelium compared to normal horizontal basal cells could explain the altered stability of the basal lamina.

More detailed analysis of the cell morphology revealed an increased distance between the plasma membrane (stained by ICAM1, intercellular adhesion molecule 1) and the nucleus in *Cxcr4*-cKO mice, resulting in a more rounded morphology (Fig. 9j, k). Cell swelling is likely caused by marked overexpression of aquaporins (AQP5 and AQP4) in the activated basal cells (Fig. 9l-–n). In addition, basal cells in the metaplastic zone showed increased expression of GJA1 (gap junction protein alpha 1, also called Connexin 43), indicating increased cell–cell communication (Fig. 9o, p). Moreover, basal cells in the metaplastic epithelium were ciliated, similar to WT horizontal basal cells, but the cilia were longer (Fig. S5d-f).

Following methimazole-induced injury, a subset of horizontal basal cells repopulates all microvillar cells and Bowman glands [64]. We therefore analyzed if basal cells in *Cxcr4*-cKO mice can still regenerate Bowman glands. Analysis of expression of AQP4 and AQP5 revealed that the number of glands in the lamina propria was markedly reduced in the D-zone (Fig. 9q, r; Fig. S5g). Moreover, staining of SOX10 showed absence of gland ducts extending throughout the epithelium (Fig. 9s). In addition, analysis of mucin secretion confirmed absence of secreting Bowman gland cells (Fig. S5h).

Together, we found that basal cells in the degenerated D-zone showed similarities to horizontal basal cells, the stem cells present in the WT olfactory epithelium. Differences in the molecular properties impaired their capacity to regenerate cells of the neuronal lineage but also Bowman gland cells.

## Discussion

During injury-induced neurogenesis in the olfactory epithelium, axon fasciculation ensures coordinated growth in the lamina propria, whereas defasciculation and rearrangement in the olfactory bulb are required to project to specific glomeruli. CXCL12/CXCR4 signaling regulates neuronal migration, cell positioning, and axon wiring during development of the zebrafish olfactory system [8], in mammalian motor neurons [11], oculomotor axons [65, 66], the enteric nervous system [67], and in retinal ganglion axons [10]. Our study demonstrates that CXCR4 is not only a developmental guidance cue but also a key regulator of tissue reconstruction, stress response, and axon pathfinding during adult injury-induced neurogenesis. The results of the study are summarized in Fig. S6.

### Regeneration of the Olfactory Epithelium in Cxcr4-cKO Mice is Initially Faster than in WT Mice

Absence of *Cxcr4* during injury-induced neurogenesis alters the expression of transcription factors known to be involved in neuronal differentiation [68–70] but also of transcription factors that have not been implicated in olfactory neurogenesis so far, such as ID4. CellOracle [27] revealed that the absence of NEUROD1, BCL11B (B cell-lymphoma/leukemia 11B), LHX2, and OLIG2 could speed up the progression through the neuronal lineage. In agreement with CellOracle analysis, expression levels of axon pathfinding molecules were altered and initial outgrowth of new axons through the lamina propria was faster in the regenerating epithelium of *Cxcr4*-cKO mice. Enhanced neuronal differentiation was also observed on a functional level, since we found improved detection of olfactory cues during the early phase of regeneration. Although most OSNs appearing at 14 dpi are not fully mature, immature OSNs have been shown to provide odor input to the olfactory bulb and successfully perform odor detection and discrimination tasks [71]. CellOracle analysis also predicted that some of the regulated transcription factors delay or block the differentiation of *Nqo1*-positive neurons located in the dorsomedial part of the epithelium.

### Axon Guidance Depends on CXCR4 Expression

The regulation of axon guidance gene expression could be a result of the observed altered expression of transcription factors. For example, LHX2 is known to regulate expression of SEMA3A [72] and ROBO2 [73]. Alternatively, the reduced expression of Gαi-coupled CXCR4 could influence axon guidance by altering cAMP levels, since expression of guidance genes in OSNs has been shown to depend on cAMP signaling [3] and CXCR4 signaling is known to reduce the activities of axonal guidance cues by regulating cAMP levels in the spinal cord [9]. Furthermore, transcriptome analysis of *Cxcr4-*cKO mice 6 weeks after injury showed greater abundance of GO terms relating to the unfolded protein response ER stress has been shown to regulate the glomerular coalescence of olfactory axons and the specificity of their projections during development by differential expression of axon guidance genes [4, 74].

### D-Zone Degeneration in Cxcr4-cKO Mice Could Depend on Axon Mistargeting

Finally, after early successful epithelial regeneration, *Cxcr4* knockout results in neural degeneration in the D-zone. OSNs located in the dorsomedial (D-zone) and ventrolateral (V-zone) region of the olfactory epithelium send their axons to the dorsal and ventral region of the olfactory bulb, respectively. Consistent with the known role of D-zone neurons in the innate response to aversive odors [60], degeneration of the D-zone in *Cxcr4*-cKO mice also caused impaired aversive behavior in response to predator odors. Cookie finding performance was indistinguishable from WT in this late phase of regeneration, consistent with previous reports showing that wiring errors and altered organization of the olfactory system have relatively minor effects on olfactory abilities of mice [75, 76]. Since staining of OSN axons in the olfactory bulb was reduced and axonal coalescence in the glomeruli of the olfactory bulb was disturbed, the absence of OSNs could be the consequence of a failure in axon pathfinding. Axons that extend beyond the target layer into the deeper layers of the olfactory bulb degenerate during development [77]. This raises the question of why only the D-zone neurons degenerate, despite CXCR4 being expressed throughout the olfactory epithelium.

The zones of the olfactory epithelium are inherently distinct, resulting in differences in cellular homeostasis that modulate the capacity of the regions to adjust to extrinsic influences. OSNs in the D-zone show differences in axon extension, which may cause different susceptibility to the alteration of axon guidance gene expression in *Cxcr4*-cKO mice. These neurons are generated earlier in development, which correlates with faster axonal extension and emergence of glomerular structures in the olfactory bulb [78]. Furthermore, D-zone neurons are more susceptible to axonal transport inhibition, since impaired neurofilament-based slow axonal transport caused by application of IDPN (3,3′-Iminodipropionitrile) results in specific D-zone degeneration in young adult rats [79]. In addition, expression of axon guidance genes depends on ER stress, and D-zone neurons tend to have higher ER stress scores than neurons from the V-zone [4].

### D-Zone Degeneration in Cxcr4-cKO Mice Could Depend on Cellular Stress

Cell death in the D-zone of regenerating *Cxcr4*-cKO mice may also occur entirely independent of the described axon pathfinding and wiring phenotype. ER stress and oxidative stress are two closely associated events and can coincide as protein folding is influenced by the redox state of the ER [80]. OSNs in the D-zone, but not in the ventral part, express NQO1, an enzyme that catalyzes the reduction of a broad range of cytotoxic quinones resulting in protection from oxidative stress [81]. Subtle changes in NQO1 have been associated with neurodegenerative diseases. For example, NQO1 is expressed in the substantia nigra and has been found to be markedly increased in the early and intermediate stages of Parkinson disease but decreases in the end stage of the disease when dopaminergic neurons die [82]. The observed degeneration of D-zone neurons during injury-induced neurogenesis in aged WT mice could therefore indicate an altered stress level as the underlying cause. Aging, eventually leading to cellular dysfunction, is driven significantly by a dysregulated unfolded protein response [83] and by dysregulation of antioxidant systems resulting in increased oxidative stress [84]. In addition, sleep deprivation, which causes oxidative stress in both brain and peripheral tissues [85], led to impaired regeneration of OSNs in the D-zone during injury-induced neurogenesis [86].

The NQO1-positive OSNs in the D-zone are also lost when animals are under long-term caloric restriction or protein restriction [87–89], although caloric and protein restriction reduce oxidative stress in most tissues. The treatments are suggested to cause an unexpected increase in oxidative stress in D-zone neurons, possibly due to NQO1 bioactivation [87]. On the other hand, long-term caloric restriction or protein restriction are conditions that cause perturbations of ER homeostasis and induce ER stress, which could as well impact cell survival or axon extension mechanisms. The increased number of NQO1-positive neurons and the increased mRNA expression of markers for mature OSNs in *Cxcr4*-cKO mice under steady-state conditions further indicate enhanced differentiation of OSNs in the absence of CXCR4. These changes were accompanied by an increased ATF5 expression in *Cxcr4*-cKO mice reflecting an enhanced stress adaption to maintain OSN homeostasis under noninjured conditions. However, during injury-induced regeneration, this adaptive response appears insufficient to sustain long-term tissue integrity. Consequently, the initial advantage of accelerated neurogenesis (14 dpi) is followed by a progressive decline of regenerative capacity, resulting in the D-zone degeneration and irreversible loss of functional olfactory epithelium (42 dpi).

### Respiratory Metaplasia Depends on Activation of Horizontal Basal Cells

The degenerated D-zone was composed of basal cells and ciliated respiratory-like cells. The basal cells showed increased IP3 receptor expression which has been shown to correlate with the appearance of respiratory barrier cells instead of sensory neurons [63]. Furthermore, basal cells in the metaplastic D-zone expressed markers related to horizontal basal cells at higher levels, while laminin expression seemed to be reduced, affecting basal lamina stability. In addition, basal cells in the metaplastic zone showed increased expression of aquaporins which correlated with a change from a flat to a rounded cellular morphology, known to occur during activation of horizontal basal cells. Apart from a failure to sustain neurogenesis, another marked difference was observed in *Cxcr4*-cKO mice with regard to the regeneration of Bowman glands. Bowman glands are derived from horizontal basal cells during injury-induced regeneration of the olfactory mucosa [64]. Basal cells in the D-zone of *Cxcr4*-cKO mice did not regenerate secretory gland cells in the lamina propria or gland ducts extending through the epithelium.

## Conclusion

Inhibitory factors, aberrant synaptogenesis, and the limited intrinsic regenerative capacity of adult neurons are obstacles to axon regeneration in the central nervous system, resulting in permanent disabilities after spinal cord injury or stroke. Our study identifies CXCR4 as a key determinant of regenerative success during injury-induced neurogenesis. Despite the faster replacement of lost neurons in *Cxcr4*-cKO mice, axon guidance and glomerular targeting were impaired, perhaps as a result of altered adaptive stress response. In *Cxcr4*-cKO mice, initial rapid regeneration was followed by long-term degeneration of the sensory epithelium, indicating that coordination and maintenance of regenerative capacity are critical for functional recovery.

## Supplementary Information

Below is the link to the electronic supplementary material.

ESM 1 Supplementary Material 1: Response of WT mice to the water control stimulus. (MOV 21.8 MB)

ESM 2 Supplementary Material 2: WT mice avoid hexanal. (MOV 20.4 MB)

ESM 3 Supplementary Material 3: *Cxcr4*-cKO mice do not avoid hexanal. (MOV 21.5 MB)

ESM 4 Supplementary Material 4 (PDF 6.50 MB)

ESM 5 Supplementary Material 5 (PDF 6.81 MB)

## Data Availability

No datasets were generated or analyzed during the current study.

## Abbreviations

OSN: Olfactory sensory neuron
ER: Endoplasmic reticulum
D-zone: Dorsomedial part of the olfactory epithelium
V-zone: Ventral part of the olfactory epithelium
UMAP: Uniform manifold approximation and projection
dpi: Days post injection
m.o.: Month of age
WT: Wild type
cKO: Conditional knockout
