# Supplementary material for: CXCR4 Coordinates Long-Term Olfactory Epithelial Regeneration and Cellular Stress Response After Injury
Source: Mol Neurobiol. 2026 Sep 23;63(1):918. doi: 10.1007/s12035-026-06215-x (PMC13601160; doi:10.1007/s12035-026-06215-x)
Supplement: Supplementary file 1 [file 12035_2026_6215_MOESM4_ESM.pdf]

Supplementary Figures

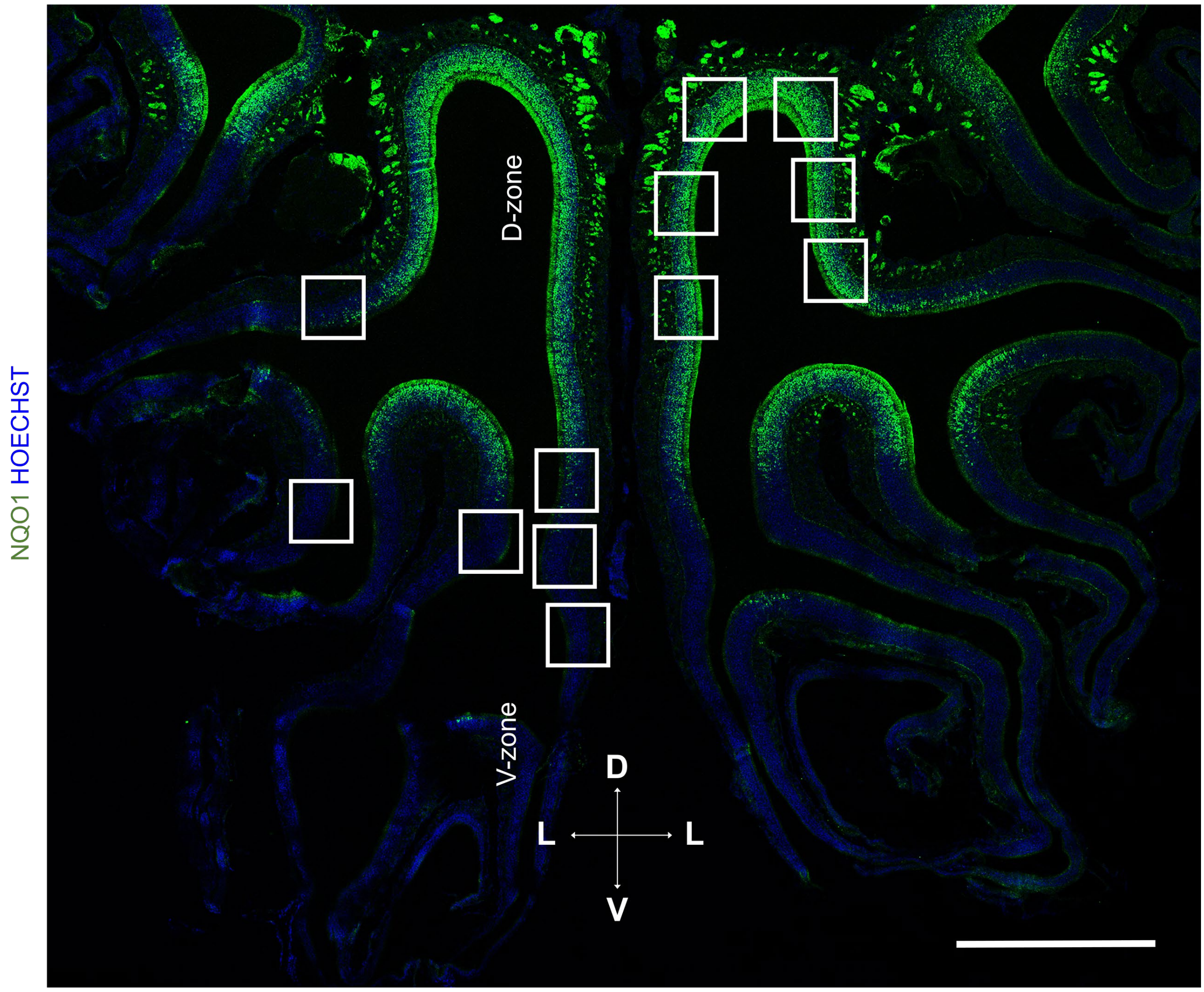

Supplementary Fig. S1

Confocal low magnification image stained with NQO1 (green) representing the dorsomedial zone (D-zone) of the WT olfactory epithelium, the ventromedial zone (V-zone) is not stained, nuclei are labeled with HOECHST (blue). D=dorsal, V=ventral, L=lateral. White boxes show the position of the quantified areas by way of example. Scale bar: 100 µm.

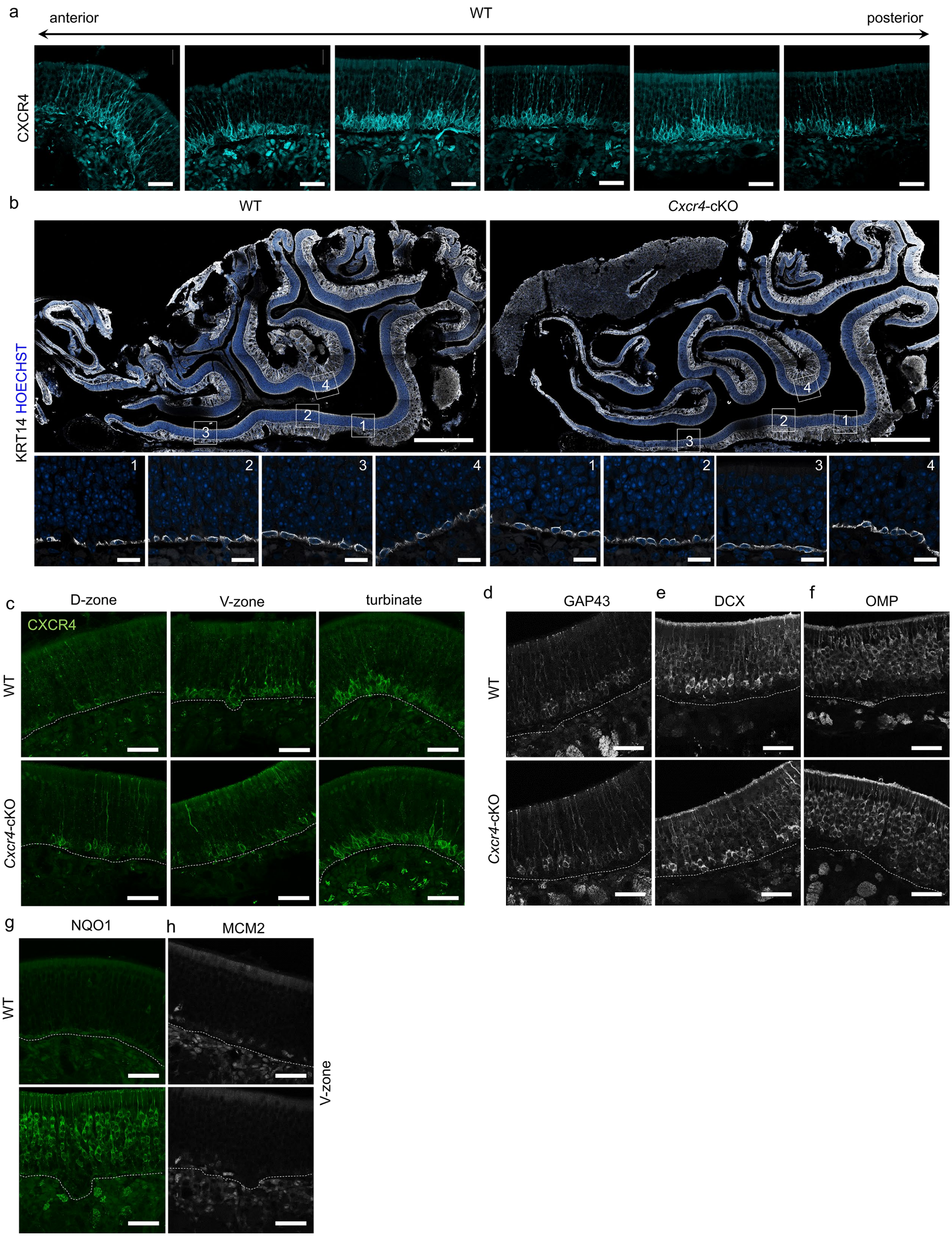

Supplementary Fig. S2

(**a**) Immunofluorescence of CXCR4 shows equal expression along the anterior-posterior axis in WT mice. (**b**) Immunofluorescent overview images of WT and *Cxcr4*-cKO mice, showing no obvious difference in KRT14-expression (white) all over the olfactory epithelium (co-

stained with HOECHST (blue)). (**c**) Comparable expression of CXCR4 in WT and *Cxcr4*-cKO mice. (**d-f**) Immunofluorescent images for GAP43 (**d**), DCX (**e**), and OMP (**f**) in the V-zone of the olfactory epithelium, showing no difference between WT and *Cxcr4*-cKO mice. (**g**) Immunofluorescence of the D-zone specific neuronal marker NQO1 showing extended labeling of neurons in the ventral part of the olfactory epithelium in *Cxcr4*-cKO mice, shown are similar position at the septum along the dorsal/ventral axis. (**h**) Immunofluorescent staining of MCM2 in the V-zone, indicating no difference between WT and *Cxcr4*-cKO mice. Scale bars: 100 µm (b overview), 10µm (b higher magnifications), 20µm (a, c, d, e, f, g, h). Dotted lines represent basal lamina.

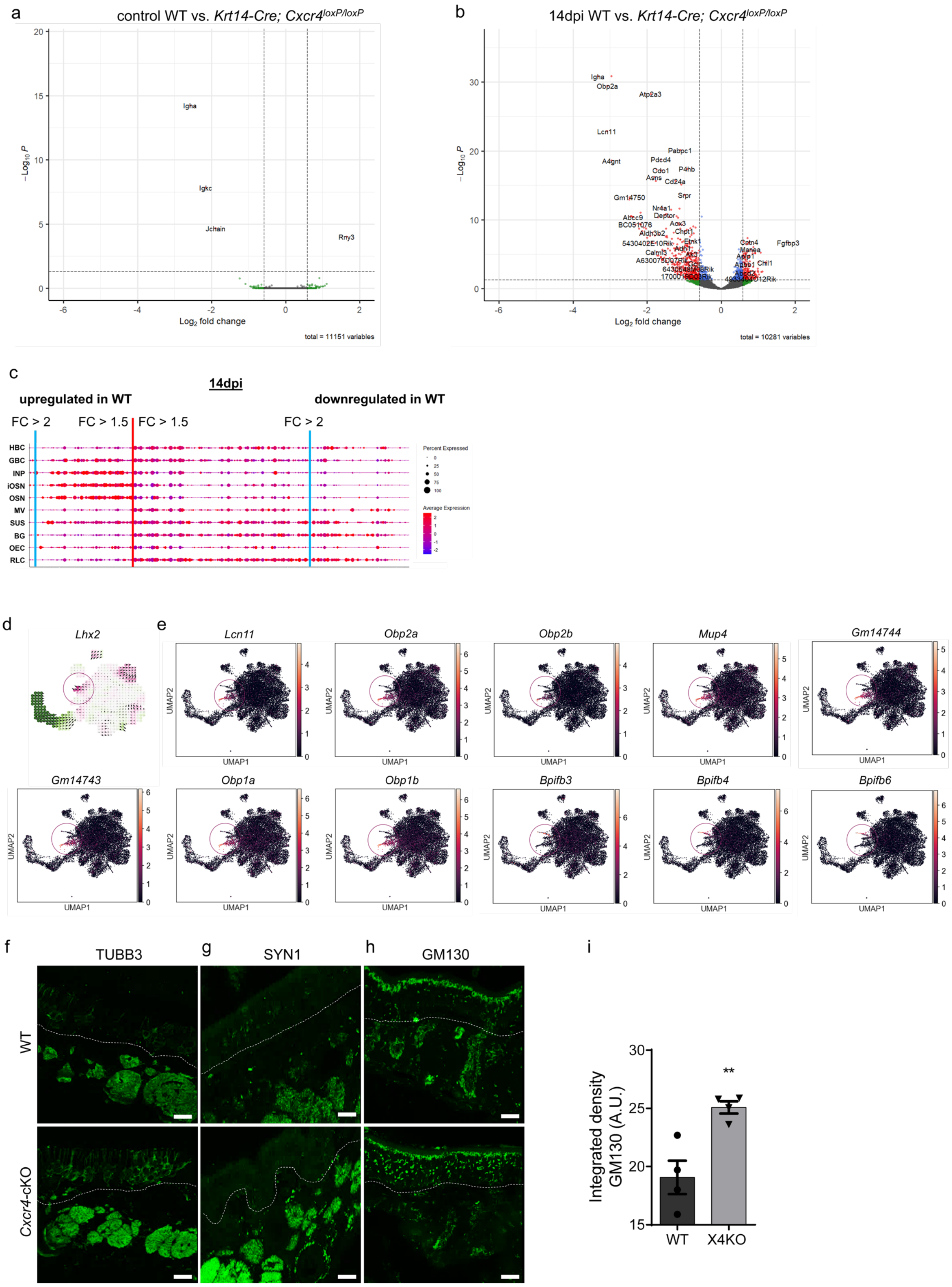

Supplementary Fig. S3

(**a, b**) Visualization of the DE analysis dataset via scatter plots with complete labeling of all genes for WT compared to *Cxcr4*-cKO mice, shown are control (**a**) and 14 dpi (**b**). (**c**) Dot Plot (from single cell RNA-seq data of adult olfactory mucosa {Wang, 2022 #299}) visualizing most

of the genes, which are regulated during early regeneration (14dpi) are expressed in the neuronal lineage. (**d**) In silico *Lhx2* perturbation using CellOracle analysis. Vector map show transitions in cell identity, color codes show the directionality of the perturbation vector to the natural differentiation vector. Positive perturbation scores (green) in immature neuronal progenitors/immature neurons indicate that reduced expression of the transcription factor promotes neuronal differentiation. Negative perturbation score (purple) of a small cluster of mature neurons is marked by purple circle. (**e**) UMAP presentations showing localization of regulated genes encoding secreted proteins. *Lcn11, Obp2a, Obp2b, Mup4, Gm14744, Gm14743, Obp1a, Obp1b, Bpibf3, Bpibf4* and *Bpibf6* are expressed in mature neurons localized in the cluster encircled in purple. (**f-h**) Immunofluorescent staining of the immature neuron marker TUBB3 (beta tubulin III) (**f**), the mature neuronal marker SYN1 (Synaptophysin 1, **g**) and the cis-Golgi marker GM130 (Golgi matrix protein 130) (**h**), labeling mature OSNs. All of them are increased in *Cxcr4*-cKO mice, demonstrating increased neuronal maturation. Dotted lines represent the basal lamina. Scale bars: 10 µm. (**i**) Quantification of immunofluorescent stainings of GM130 in the neuronal layer of the epithelium, excluding the apical sustentacular cells and the lamina propria, in WT and *Cxcr4*-cKO mice (n=3 animals per group, Student's t-test, error bars represent SEM, **$p < 0.01$).

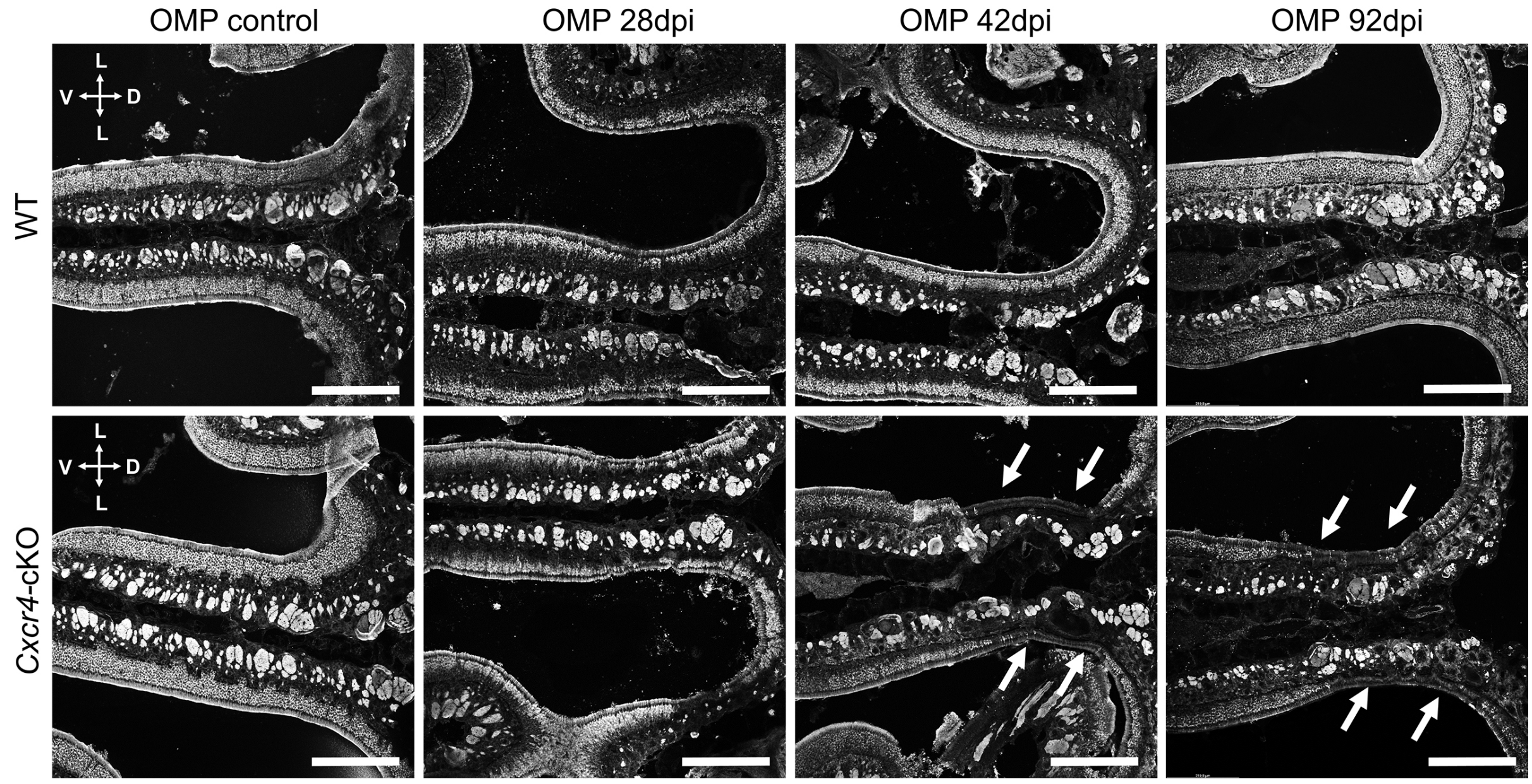

Supplementary Fig. S4

CXCR4 depletion leads to loss of D-zone neurons. Immunofluorescence labeling of OMP. There is no difference in OMP staining between uninjured WT and *Cxcr4*-cKO controls. At 28dpi, OMP labeling appeared similar in WT and *Cxcr4*-cKO mice. At 42dpi, the D-zone in WT mice is largely regenerated, although the neuronal layer continued to increase in thickness until 92 dpi. The D-zone in *Cxcr4*-cKO mice showed signs of degeneration (42 dpi, 92 dpi, see arrows). L=lateral; V=ventral, D=dorsal, arrows point to degenerated parts. Scale bars 100µm.

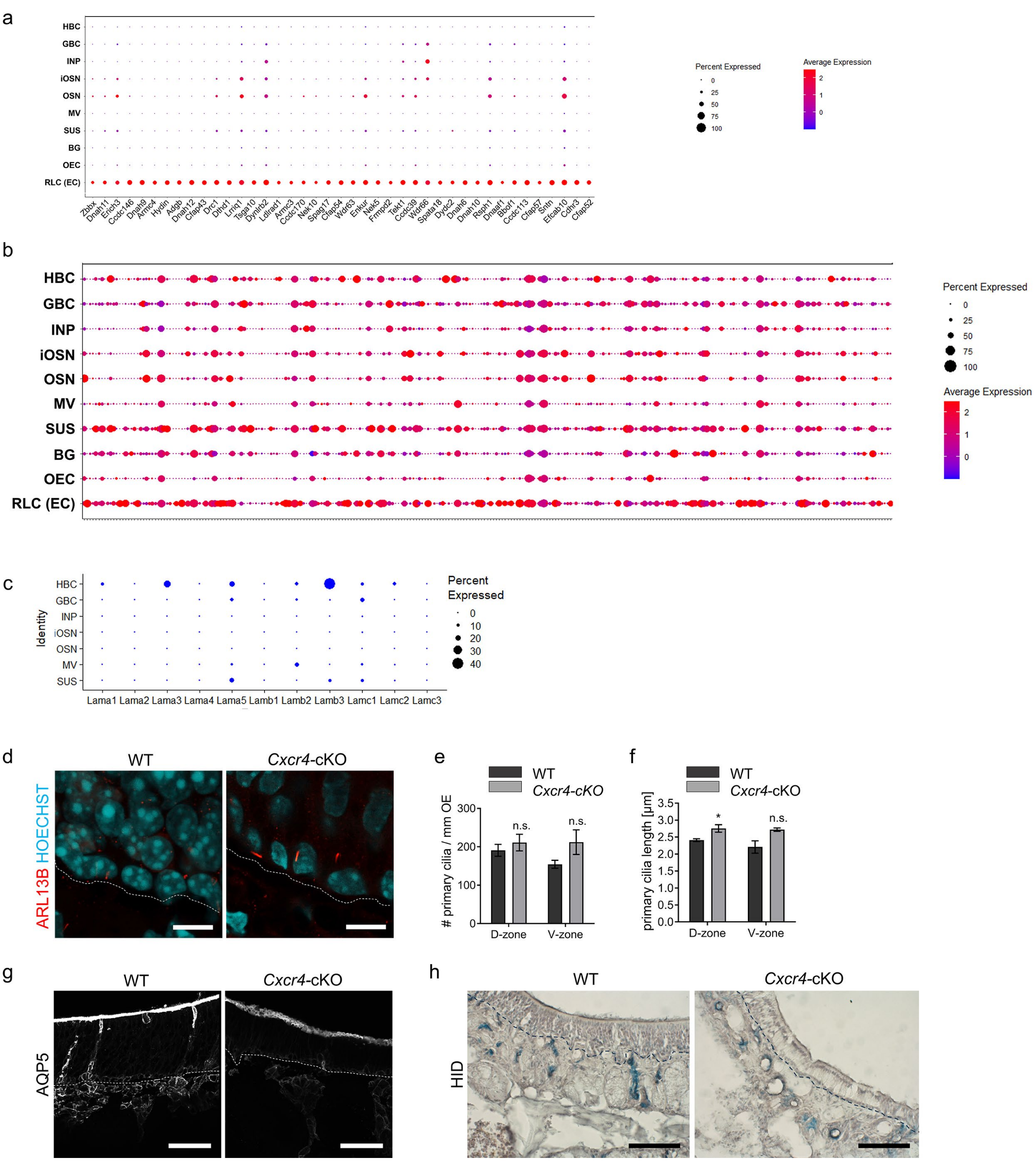

Supplementary Fig. S5

(**a**) Dot Plot visualizing localization of marker genes of the respiratory epithelium in the olfactory mucosa. Marker genes are derived from single cell RNA-seq data of ciliated airway epithelial cells of the human lung {Everman, 2019 #400}, cell types of the olfactory mucosa are assigned based on single cell RNA-seq data {Wang, 2022 #299}. (**b**) Dot Plot visualizing localization of marker genes of the respiratory epithelium derived from the Monarch Initiative {Putman, 2023 #399}. The majority of non-ubiquitous genes is expressed by RLCs. The size of the dots represents the relative proportion of cells expressing the gene and the color indicates

the average expression level. (**c**) Dot plot showing expression of laminin genes in a published single cell RNA-seq data set of the olfactory epithelium {Wang, 2022 #299}. Laminins are mostly expressed by horizontal basal cells. (**d**) Immunofluorescent staining for ARL13b (red) and HOECHST (blue). In *Cxcr4*-cKO mice, primary cilia of basal cells were longer compared to WT mice. (**e, f**) Quantification of cilia number (**e**) and cilia length (**f**) of basal cells in the olfactory epithelium. (**g**) Immunofluorescent labeling for AQP5 in the olfactory mucosa. AQP5 was expressed by Bowman glands in the lamina propria, gland ducts extending through the epithelium, and the apical membrane of sustentacular cells. In *Cxcr4*-cKO mice, Bowman glands were almost absent in the D-zone at 42dpi. (**h**) HID (High Iron Diamine) Alcian Blue staining at 42dpi showing strongly reduced staining of Bowman glands in *Cxcr4*-cKO mice. Sulfated acidic mucus is stained brownish black, carboxylated sialic acid mucus is stained blue. HBC: horizontal basal cell; GBC: globose basal cell; INP: immediate neuronal precursor; iOSN: immature olfactory sensory neuron; OSN: olfactory sensory neuron; MV: microvillar cell; SUS: sustentacular cell; RLC: respiratory-like cells; EC: ependymal cells. Dotted lines represent the basal lamina. Scale bars 5 µm (d), 20 µm (g), 50 µm (h).

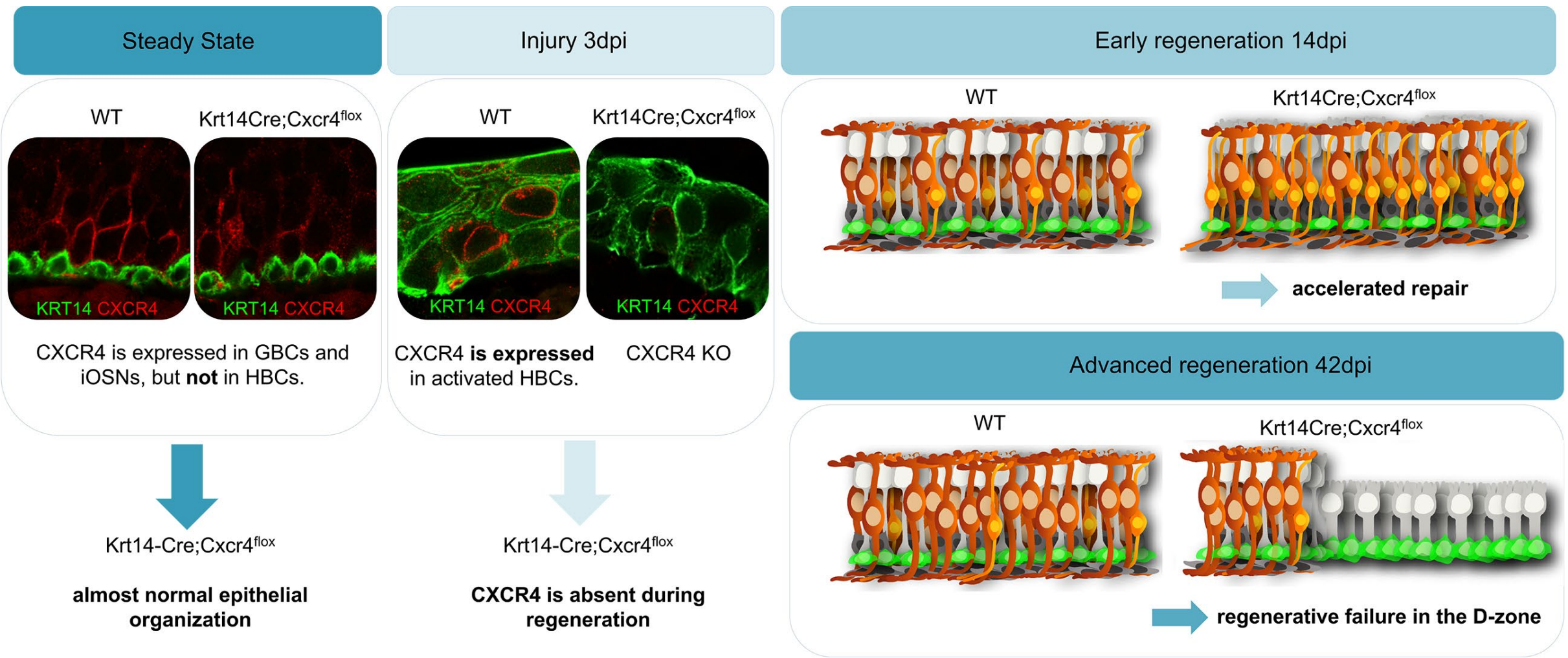

Supplementary Fig. S6

We analyzed injury-induced neurogenesis in the olfactory epithelium of *Cxcr4*-cKO mice, where *Cxcr4* was deleted in horizontal basal cells using a *Krt14*-Cre line.

Steady state: At steady state conditions, CXCR4 is expressed by globose basal cells, immediate neuronal progenitors and immature neurons; KRT14 is expressed by quiescent horizontal basal cells. CXCR4 expression was not altered in *Krt14*-Cre;*Cxcr4*^{LoxP} mice. The olfactory epithelium of *Krt14*-Cre;*Cxcr4*^{LoxP} mice was similar to the WT epithelium.

Injury 3 dpi: 3 days after methimazole-induced injury (3dpi), the olfactory epithelium is destroyed, KRT14-positive horizontal basal cells became activated to regenerate the olfactory epithelium. These activated horizontal basal cells start to express CXCR4 when rebuilding cells of the neuronal lineage. CXCR4 is not expressed by activated horizontal basal cells in *Krt14*-Cre;*Cxcr4*^{LoxP} (*Cxcr4*-cKO) mice.

Early regeneration 14 dpi: The number of immature and mature differentiated neurons and the staining of their axons in the lamina propria was increased in *Cxcr4*-cKO mice. *Cxcr4*-cKO had a better sense of smell. However, axon targeting in the olfactory bulb was impaired.

Advanced regeneration 42 dpi: While the WT epithelium was almost completely regenerated at 42dpi, olfactory neurons were lost in the D-zone of *Cxcr4*-cKO mice, which caused reduced avoidance of the D-zone specific aversive odorant hexanal. Cells of the knockout epithelium showed an increased stress response. Basal cells of the degenerated D-zone were different from WT horizontal basal cells. Apart from generating respiratory-like cells instead of neurons, Bowman glands were not regenerated in the D-zone. The overall activity level of the basal cells was increased.

Supplementary table S1

PRIMARY ANTIBODIES	HOST SPECIES	COMPANY, ORDER NUMBER	DILUTION
Acetylated tubulin	mouse	Sigma, T6793	1:500
ADCY3	rabbit	Santa Cruz, sc-588	1:500
AQP4	rabbit	Genetex, GTX133151	1:200
AQP5	rabbit	Millipore, 178615	1:200
ARL13B	mouse	Neuromab, n295b/66, 75-287	1:200
Caspase-3	rabbit	Cell Signaling, 9664S	1:100
CNGA2	mouse	Alomone labs, APC-045	1:200
CX43	mouse	Thermo Fisher Scientific, 35-5000	1:200
CXCR4 (N-term), 2B11	rat	Affymetrix, 14-9991-82	1:500
CXCR4 (C-term)	rabbit	Self-made	1:100
DCX	guinea pig	Synaptic Systems, 326 004	1:200
GAP43	mouse	Millipore MAB347	1:100
GFAP	guinea pig	Synaptic Systems, 173004	1:300
GM130	mouse	BD Biosciences, 610822	1:200
4-HNE	mouse	R&D Systems, MAB-3249	1:100
ICAM-1	goat	R&D Systems, AF796	1:300
IP3R	mouse	BD Biosciences, 610312	1:100
KRT5	rabbit	Convance, PRB-160P)	1:500
KRT14	rabbit	Genetex, GTX104 124	1:100
LAMININ alpha1	rabbit	Sigma, L9393	1:300
LHX2	rabbit	Genetex, GTX129241	1:200
LSD-1, KDM1A	rabbit	Abcam, ab129195	1:200
MARCKS	rabbit	Proteintech, 20661-1	1:50
MCM2 (BM-28)	mouse	BD Biosciences, 610700	1:200
NQO1	goat	Biorbyt, Orb18366	1:200
OMP	goat	WAKO, 544-1001	1:500
p-PERK	rabbit	Bioss, bs-3330-R-TR	1:100
ROBO2	goat	R&D Systems, AF3147	1:200
SOX2	goat	R&D Systems, AF2018	1:200
SOX10	goat	R&D Systems, AF2864	1:200
SYN1	guinea pig	Synaptic Systems, 101 004	1:200
SDC1	rat	BD Pharmingen, 533712	1:100
TUBB3	rabbit	Biolegend, PRB-435P	1:500
UCHL1, PGP9.5	rabbit	Proteintech, 14730-1	1:100

Supplementary table S2

SECONDARY ANTIBODIES	COMPANY, ORDER NUMBER	DILUTION
Alexa Fluor® 488 donkey anti-goat	Thermo Fisher Scientific, A-11055	1:500
Alexa Fluor® 488 donkey anti-rabbit	Thermo Fisher Scientific, A-21206	1:500
Alexa Fluor® 488 donkey anti-rat	Thermo Fisher Scientific, A-21208	1:500
Alexa Fluor® 568 donkey anti-goat	Thermo Fisher Scientific, A-11057	1:500

Alexa Fluor® 568 donkey anti-guinea pig	Jackson Immuno Research, 706-575-148	1:500
Alexa Fluor® 568 donkey anti-mouse	Thermo Fisher Scientific, A-11004	1:500
Alexa Fluor® 568 donkey anti-rabbit	Thermo Fisher Scientific, A-10042	1:500
Alexa Fluor® 647 donkey anti-goat	Thermo Fisher Scientific, A-21447	1:500
Alexa Fluor® 647 donkey anti-guinea pig	Jackson Immuno Research, 706-605-148	1:500
Alexa Fluor® 647 donkey anti-rabbit	Thermo Fisher Scientific, A-31573	1:500
Alexa Fluor® 647 donkey anti-rat	Jackson Immuno Research, 712-605-153	1:500
Hoechst3342	Thermo Fisher Scientific, 62249	1:500
Anti-goat IgG, HRP-linked antibody	Santa Cruz, sc-2056	1:500
Anti-mouse IgG, HRP-linked antibody	NEB, 7076	1:500
Anti-rabbit IgG, HRP-linked antibody	NEB, 7074	1:500
