# Supplementary material for: CXCR4 Coordinates Long-Term Olfactory Epithelial Regeneration and Cellular Stress Response After Injury
Source: Mol Neurobiol. 2026 Sep 23;63(1):918. doi: 10.1007/s12035-026-06215-x (PMC13601160; doi:10.1007/s12035-026-06215-x)

Uncropped images Figure 1

Fig. 1i Fig. 1j

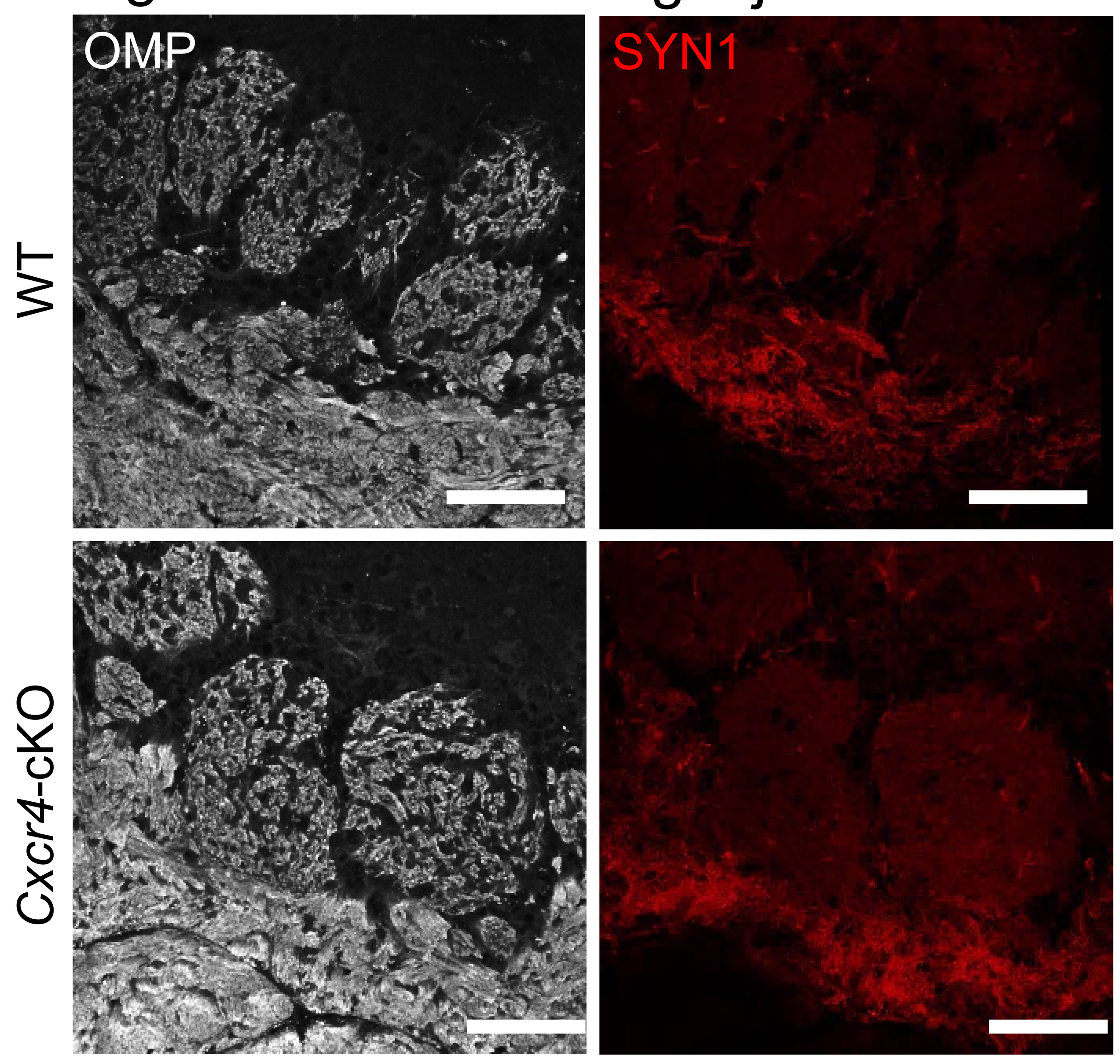

Fig. 1q

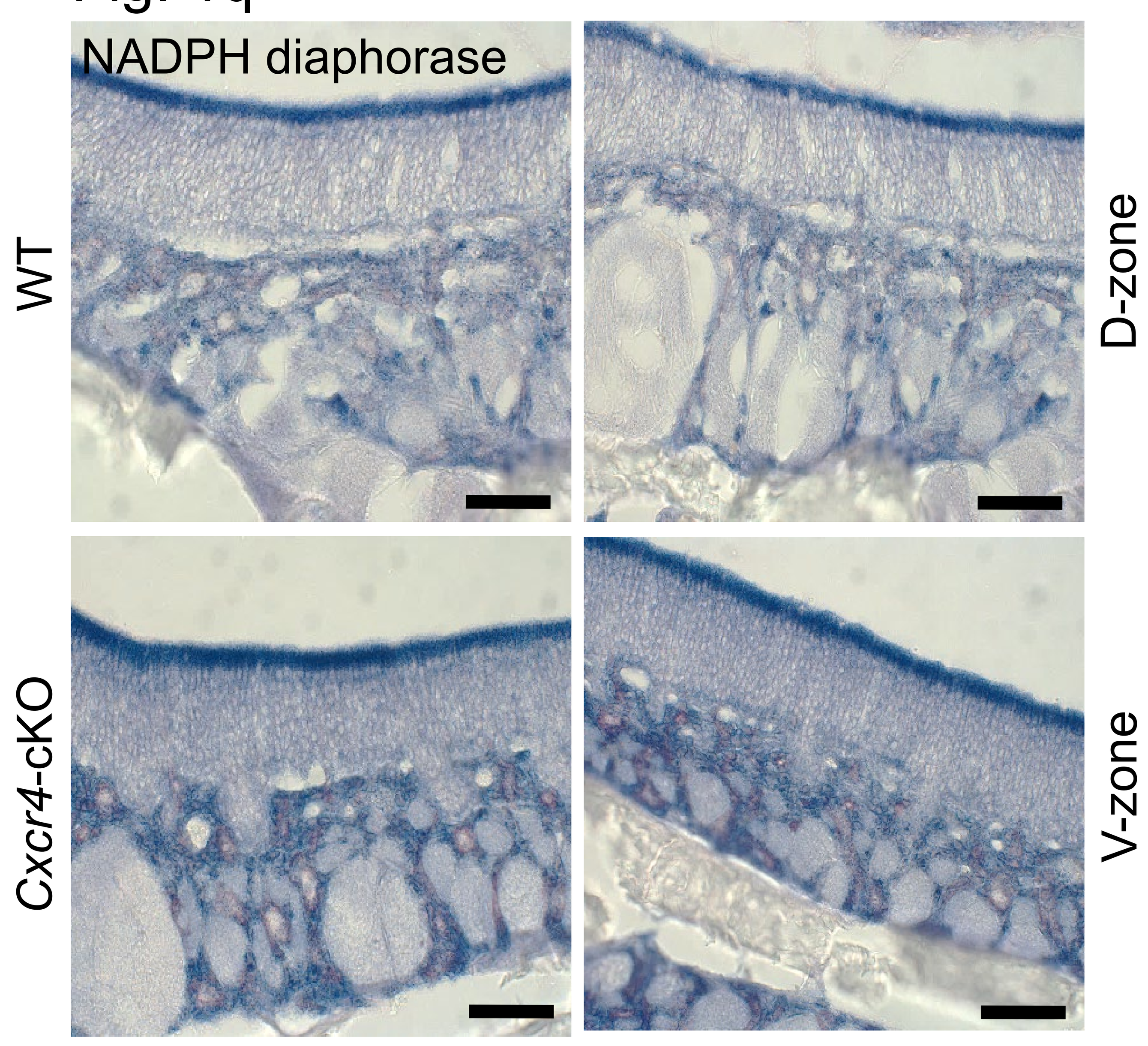

Fig. 1r

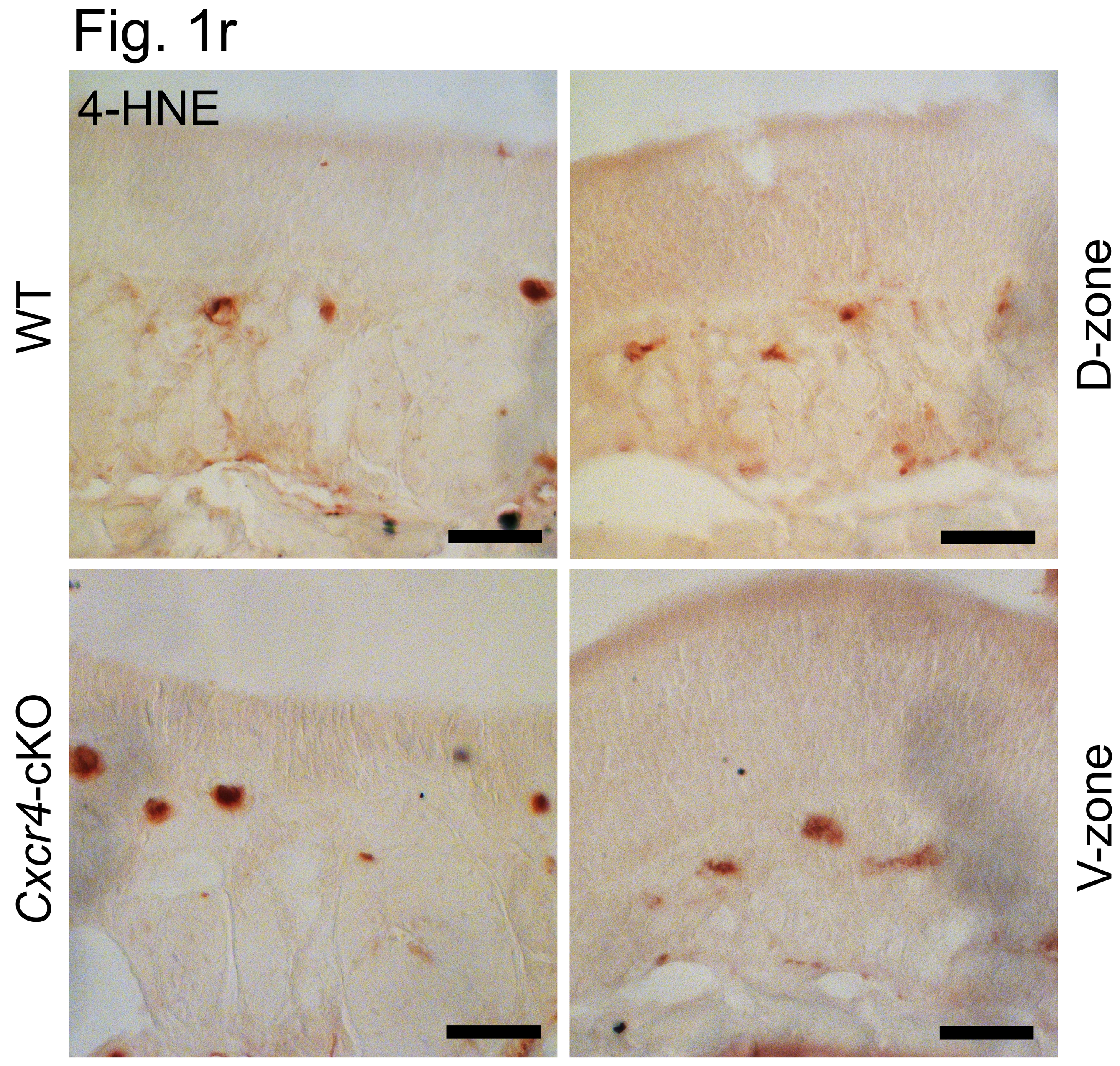

Uncropped images Figure 2

Fig. 2b

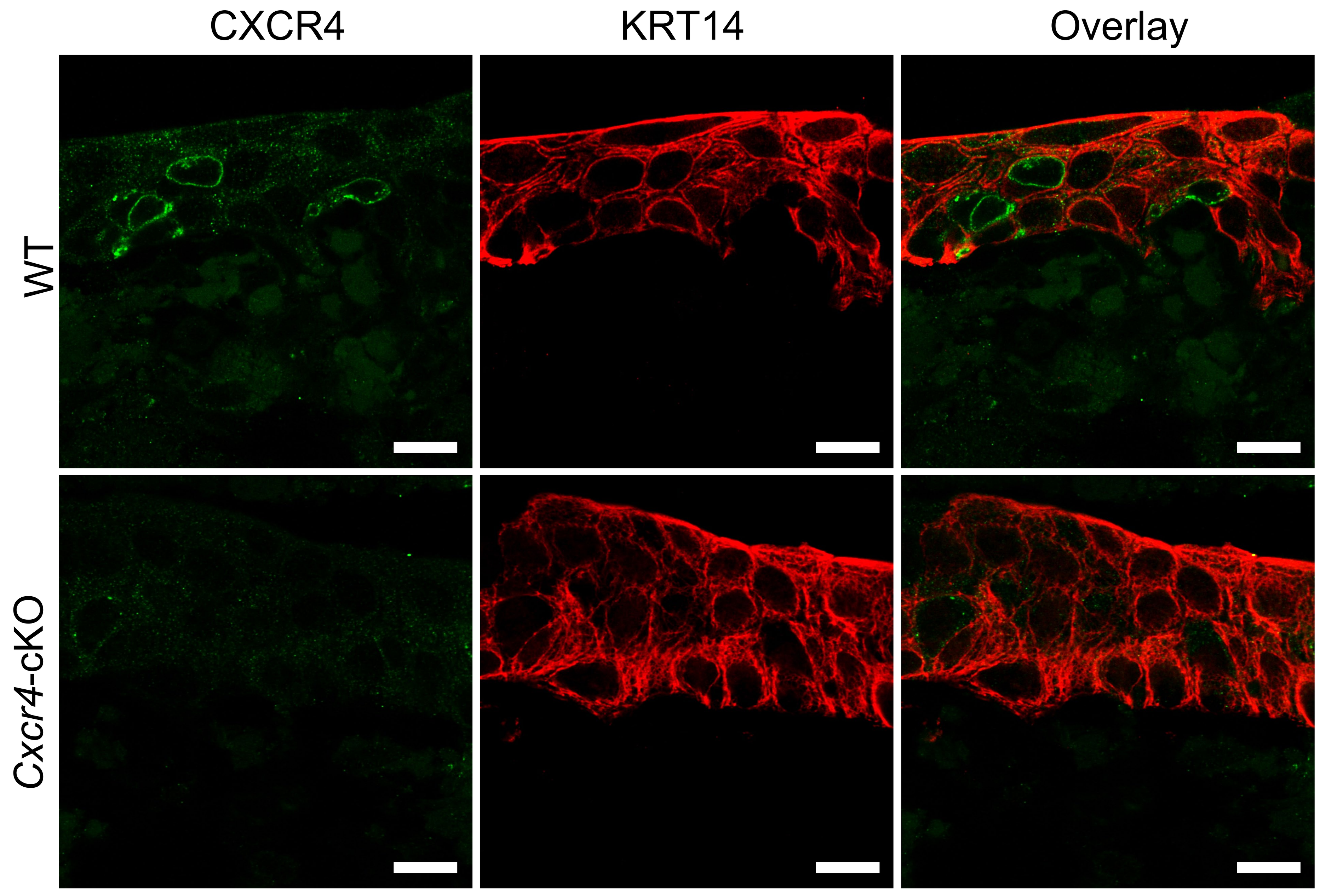

Uncropped images Figure 4

Fig. 4g Fig. 4h Fig. 4i

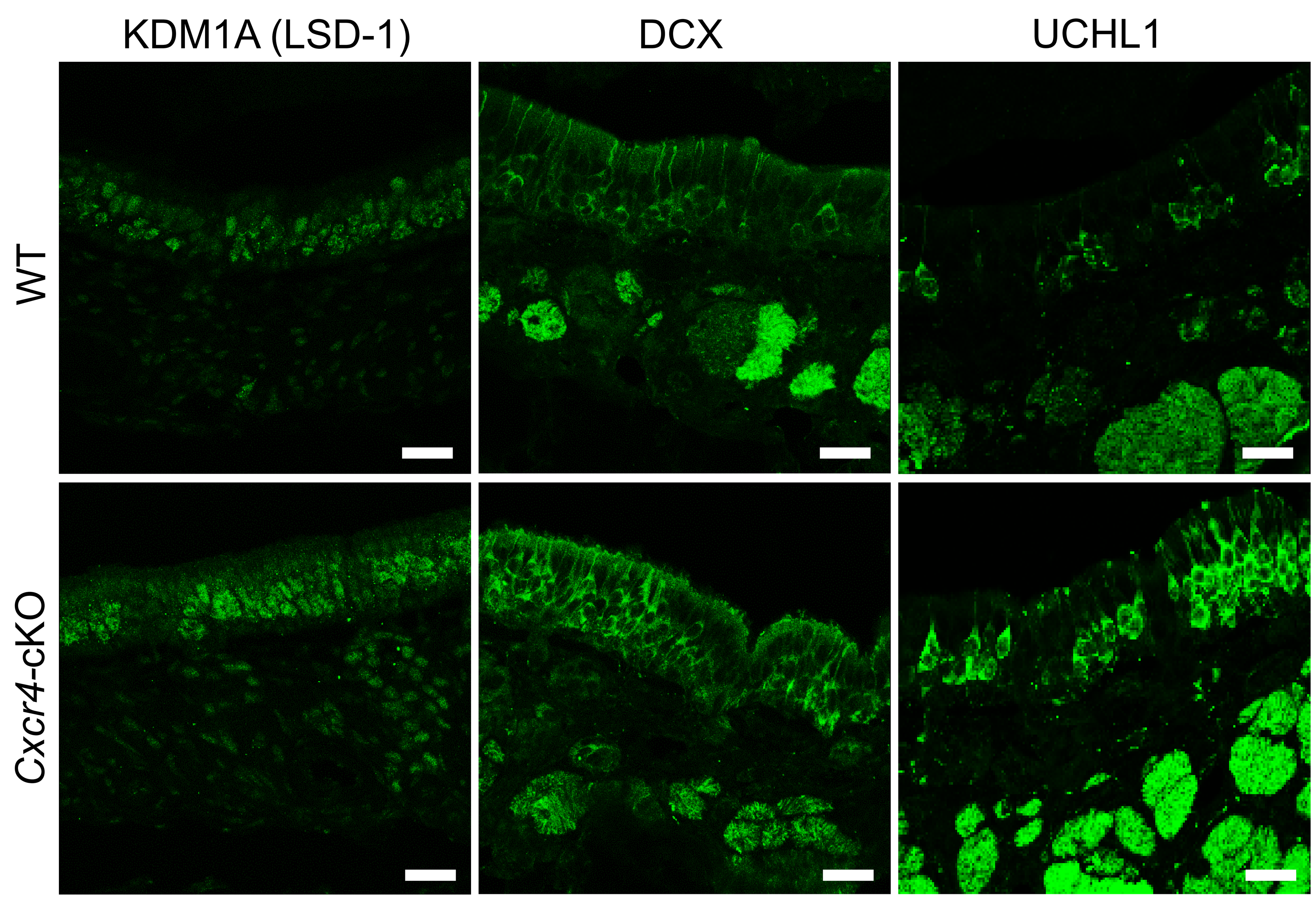

Uncropped images Figure 5

Fig. 5d

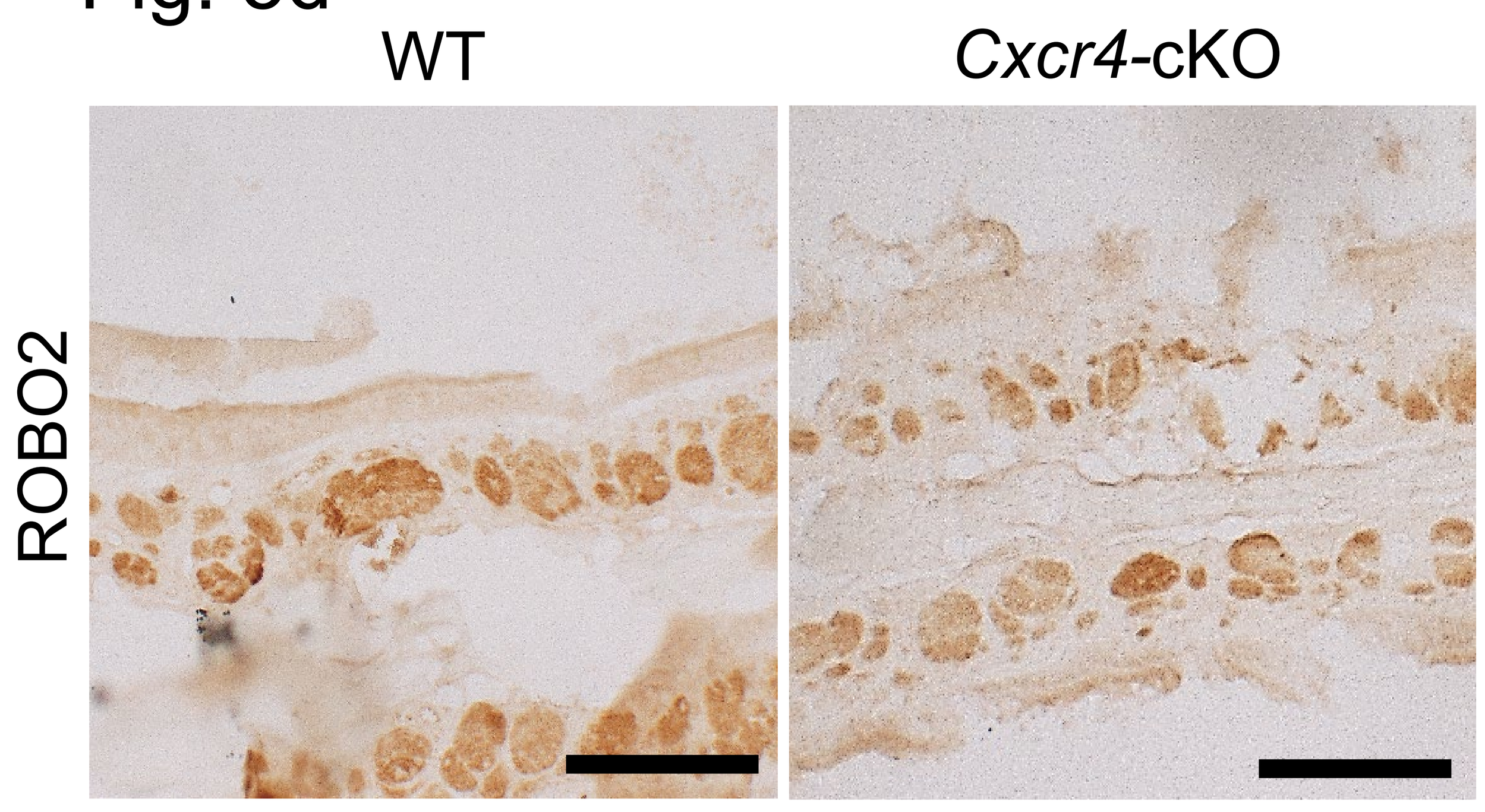

Fig. 5j

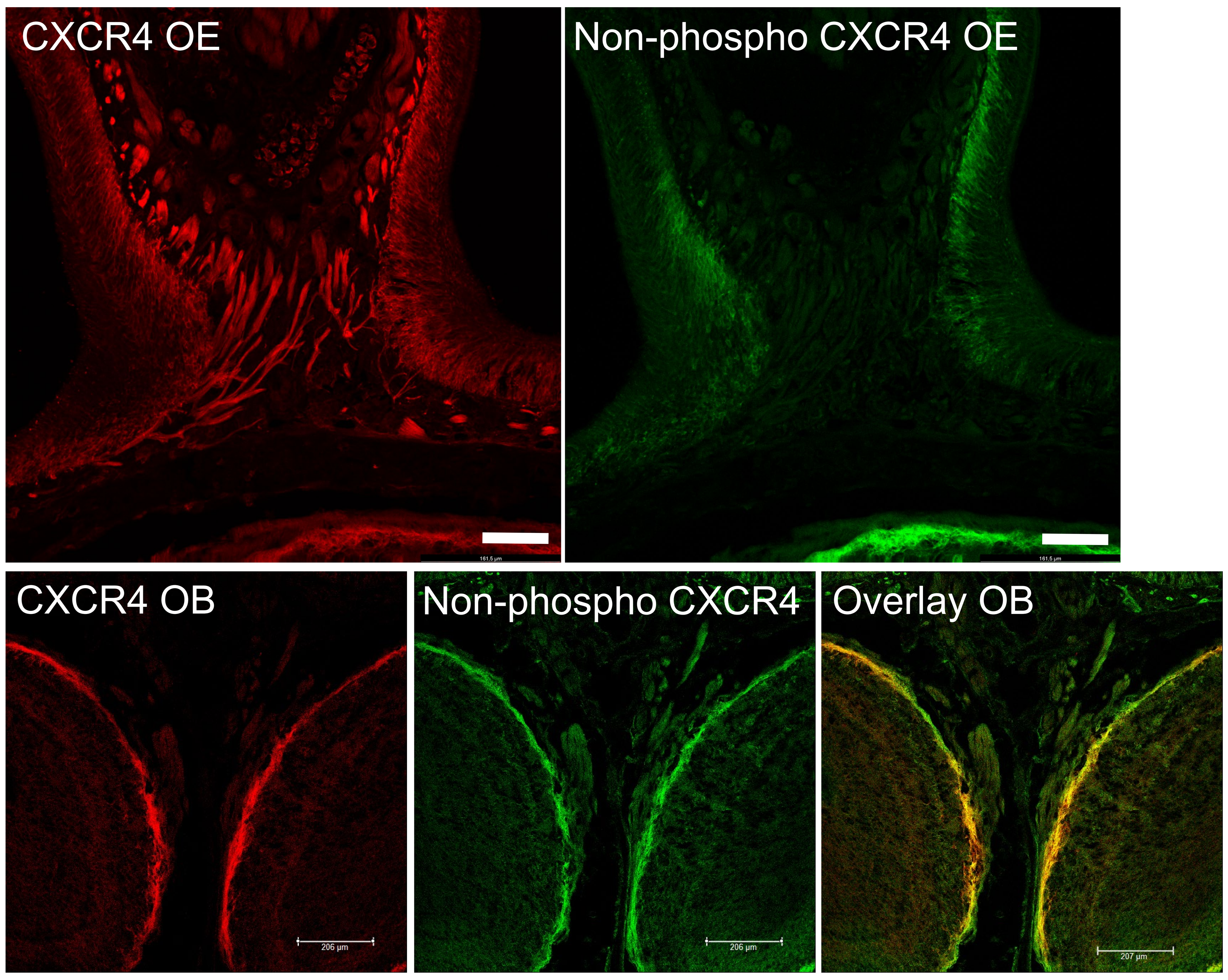

Uncropped images Figure 7

Fig. 7b

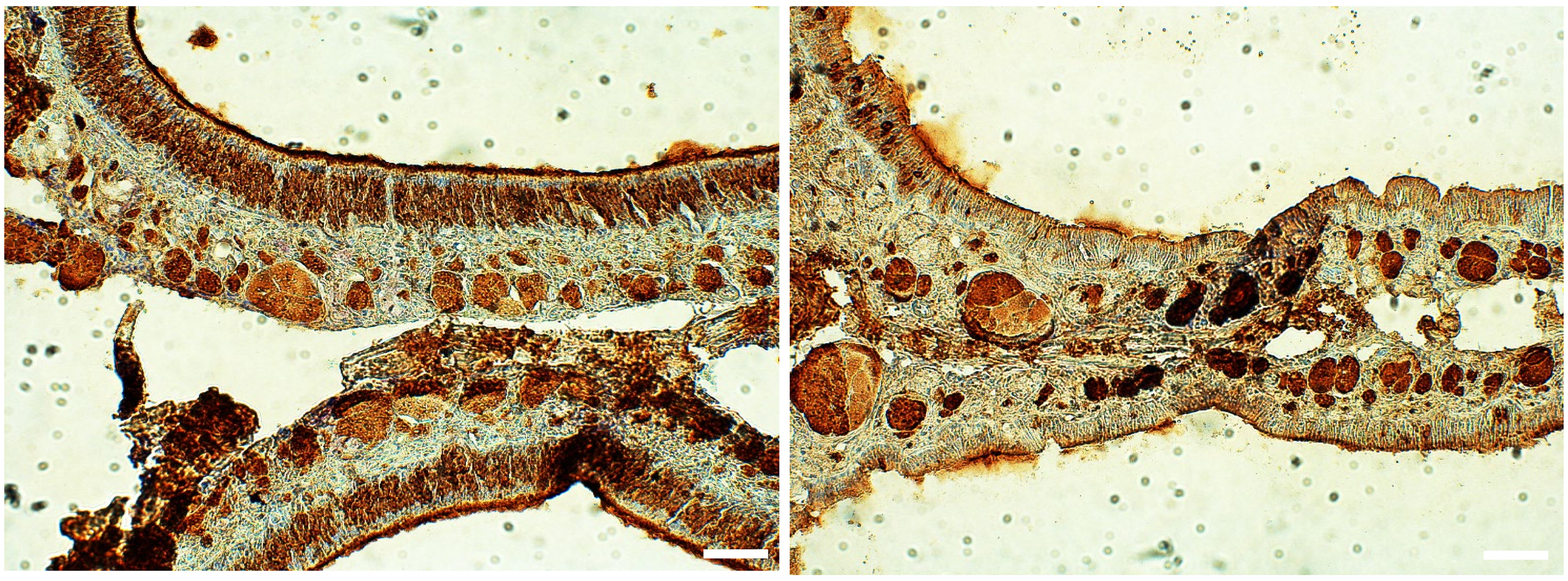

Uncropped images Figure 8

Fig. 8d

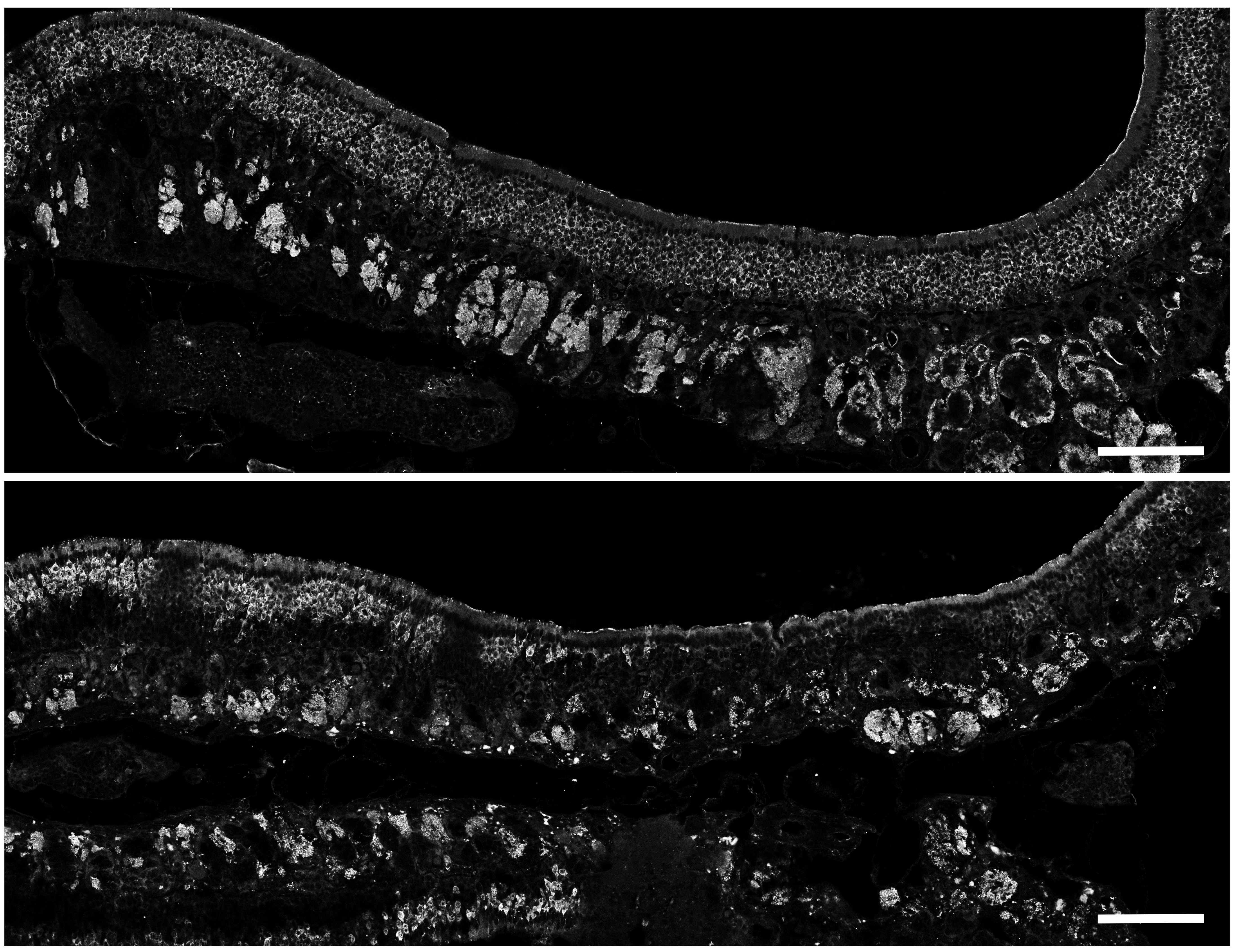

Uncropped images Figure 9

Fig. 9c

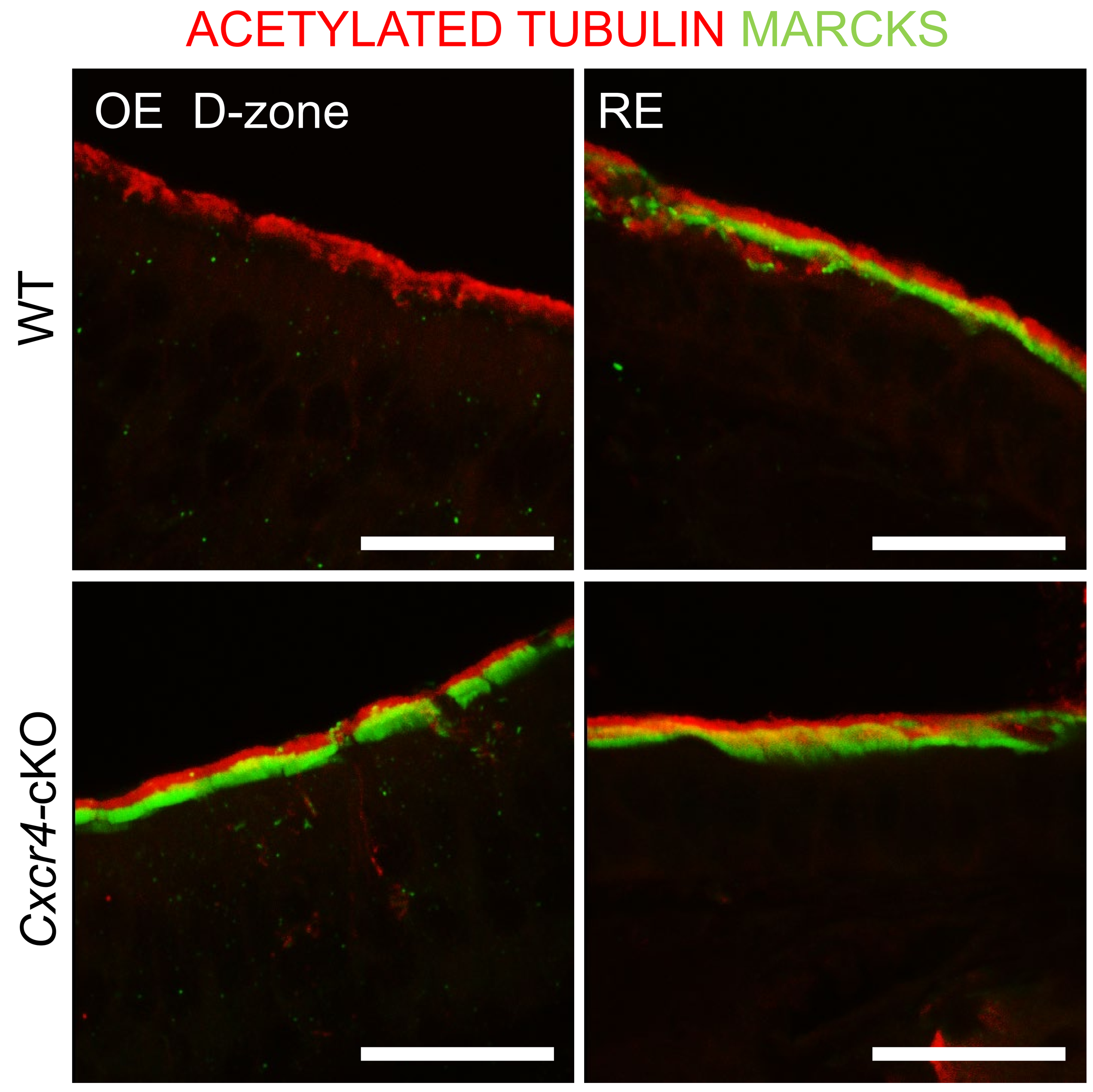

Fig. 9d

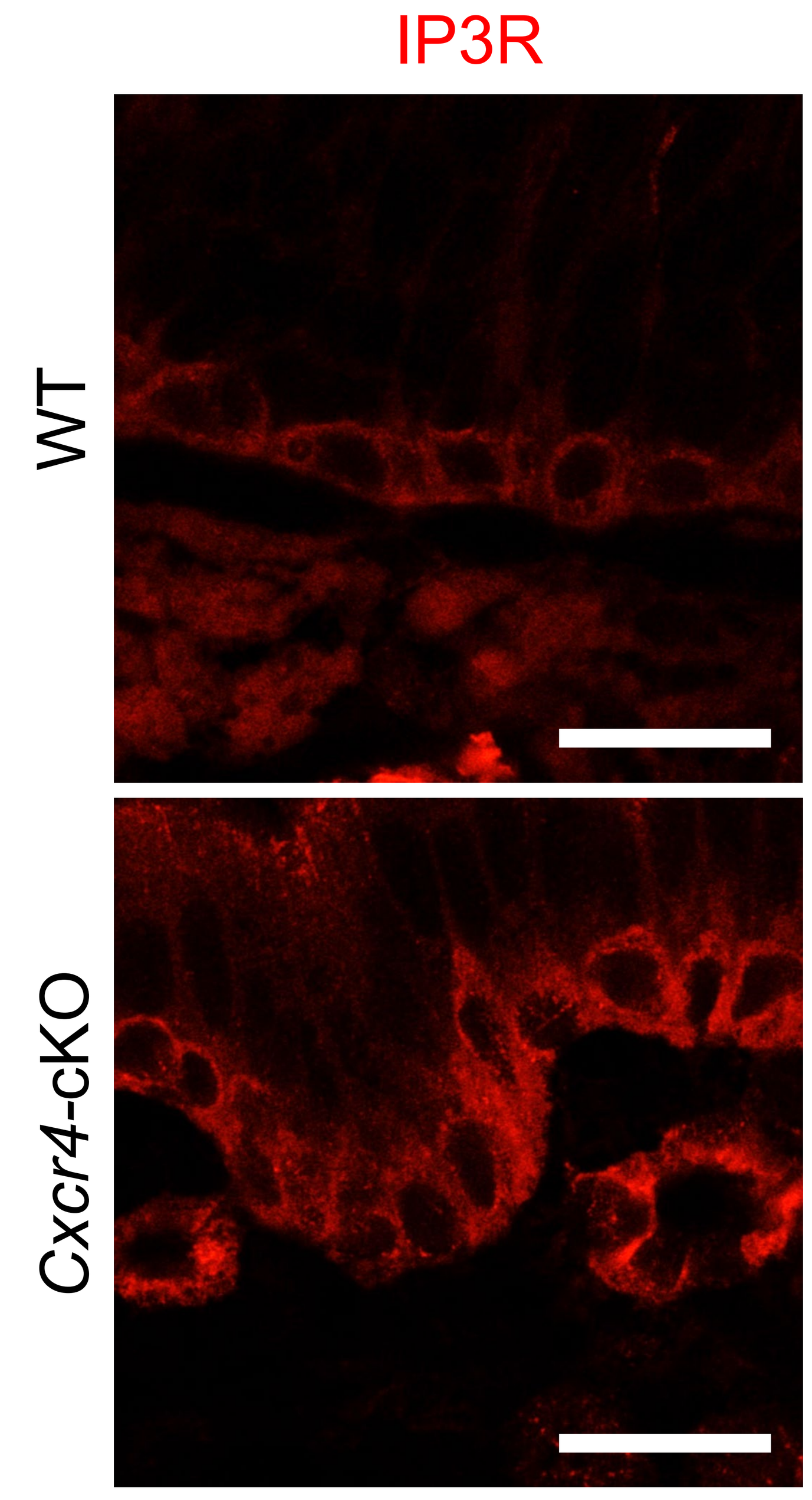

Fig. 9f

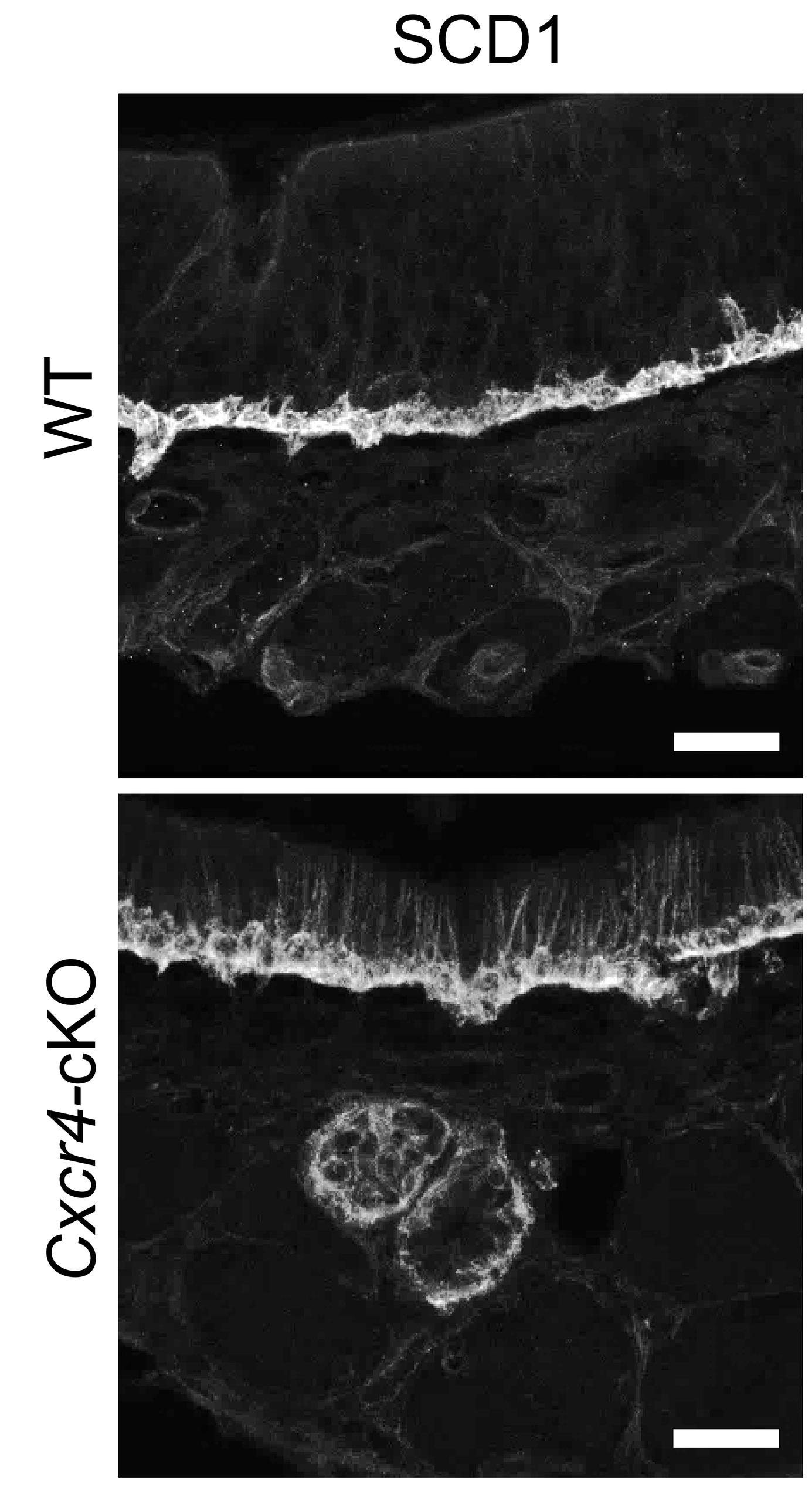

Fig. 9g

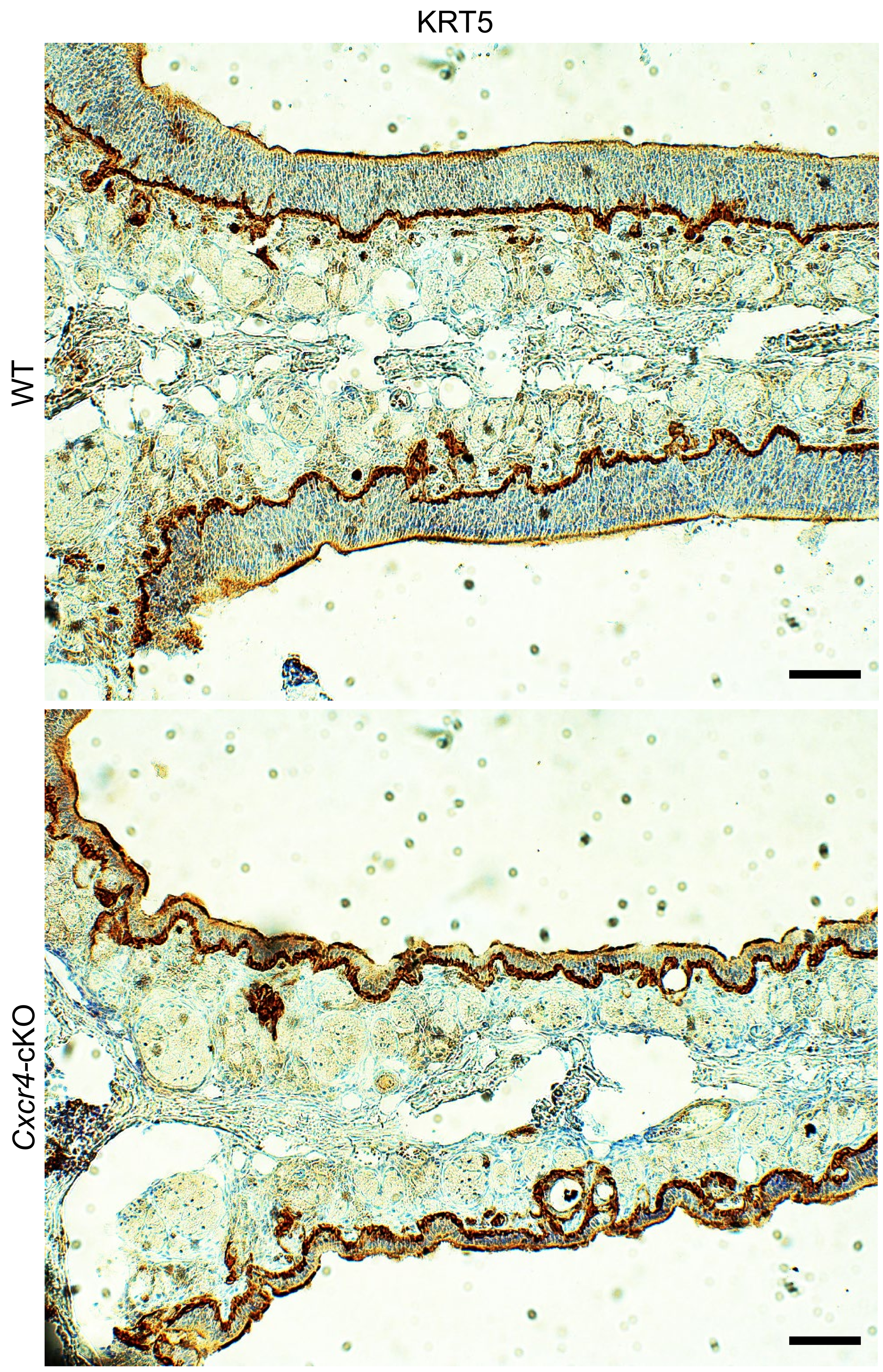

Fig. 9i

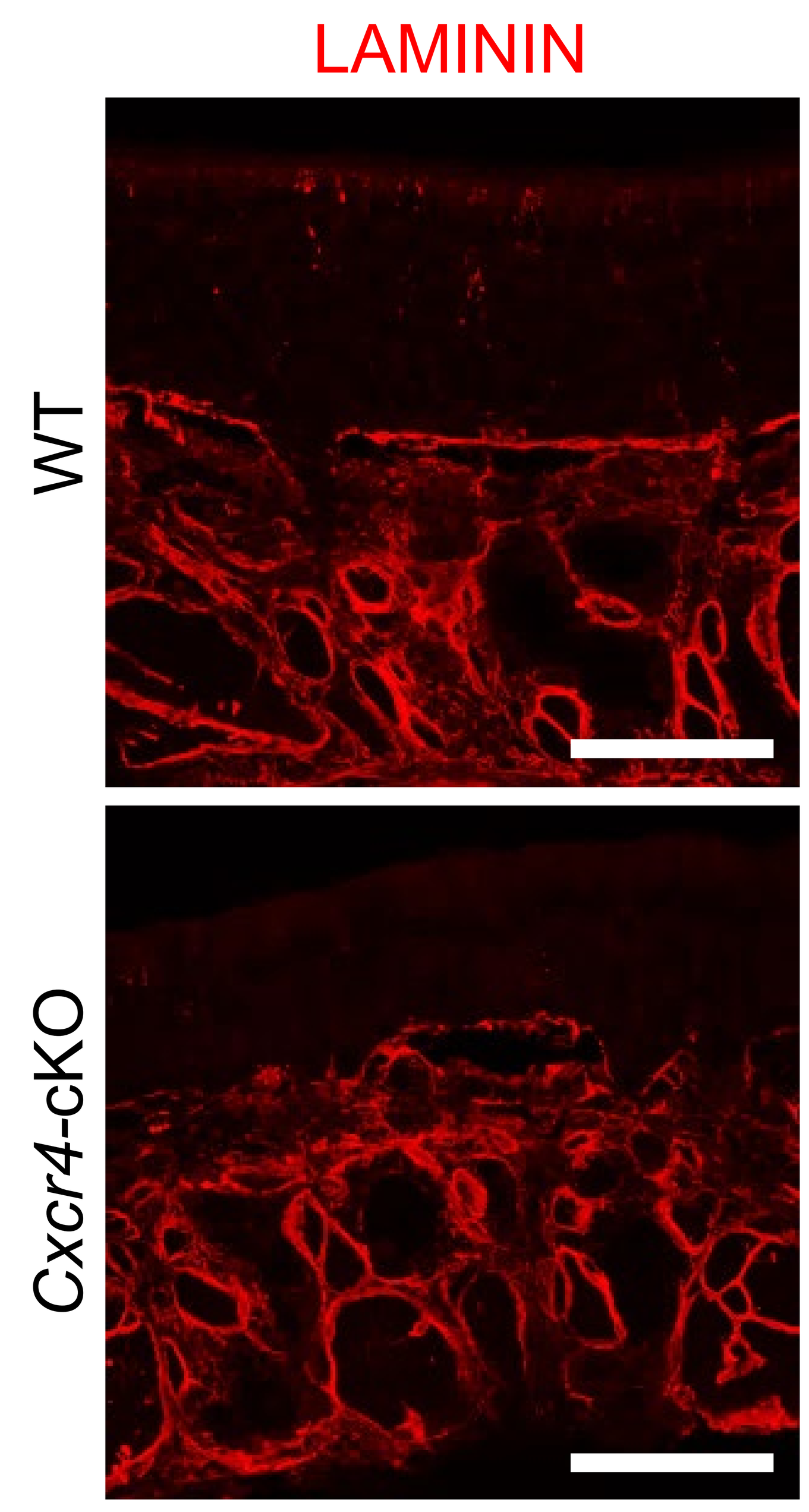

Fig. S3h

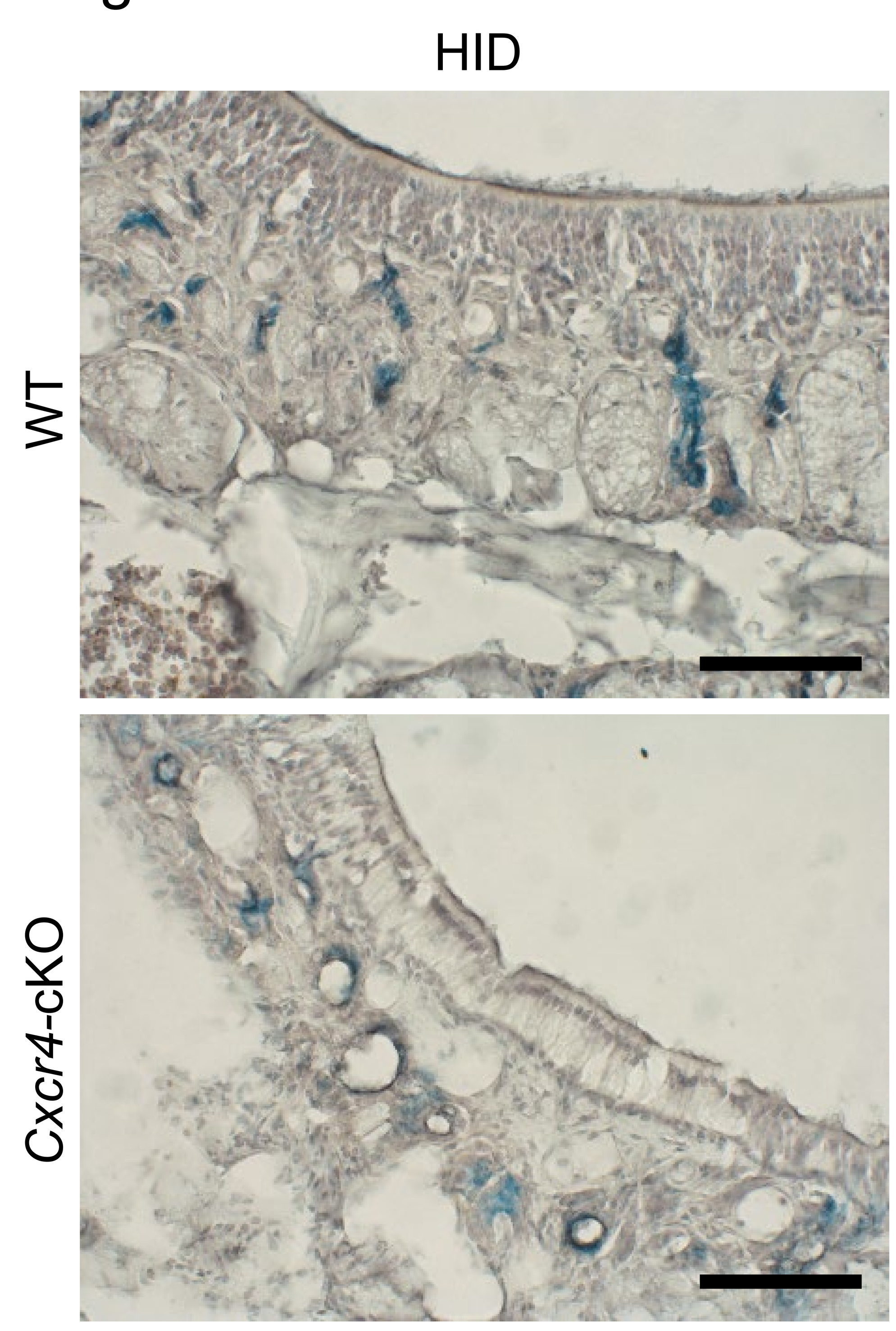

Supplement: Supplementary file 2 [file 12035_2026_6215_MOESM5_ESM.pdf]
